# Epigenetic regulation of seed-specific gene expression by DNA methylation valleys in castor bean

**DOI:** 10.1186/s12915-022-01259-6

**Published:** 2022-03-01

**Authors:** Bing Han, Di Wu, Yanyu Zhang, De-Zhu Li, Wei Xu, Aizhong Liu

**Affiliations:** 1 Germplasm Bank of Wild Species, Kunming Institute of Botany, Chinese Academy of Sciences, Kunming, 650201 Yunnan China; 2 Key Laboratory of Economic Plants and Biotechnology, Yunnan Key Laboratory for Wild Plant Resources, Kunming Institute of Botany, Chinese Academy of Sciences, Kunming, 650201 China; 3 University of Chinese Academy of Sciences, Beijing, 100049 China; 4 Key Laboratory for Forest Resources Conservation and Utilization in the Southwest Mountains of China, Ministry of Education, Southwest Forestry University, Kunming, 650224 China

**Keywords:** Seed-specific genes, DNA methylation valleys, Histone modifications, Enhancer, Castor bean

## Abstract

**Background:**

Understanding the processes governing angiosperm seed growth and development is essential both for fundamental plant biology and for agronomic purposes. Master regulators of angiosperm seed development are expressed in a seed-specific manner. However, it is unclear how this seed specificity of transcription is established. In some vertebrates, DNA methylation valleys (DMVs) are highly conserved and strongly associated with key developmental genes, but comparable studies in plants are limited to *Arabidopsis* and soybean. Castor bean (*Ricinus communis*) is a valuable model system for the study of seed biology in dicots and source of economically important castor oil. Unlike other dicots such as *Arabidopsis* and soybean, castor bean seeds have a relatively large and persistent endosperm throughout seed development, representing substantial structural differences in mature seeds. Here, we performed an integrated analysis of RNA-seq, whole-genome bisulfite sequencing, and ChIP-seq for various histone marks in the castor bean.

**Results:**

We present a gene expression atlas covering 16 representative tissues and identified 1162 seed-specific genes in castor bean (*Ricinus communis*), a valuable model for the study of seed biology in dicots. Upon whole-genome DNA methylation analyses, we detected 32,567 DMVs across five tissues, covering ~33% of the castor bean genome. These DMVs are highly hypomethylated during development and conserved across plant species. We found that DMVs have the potential to activate transcription, especially that of tissue-specific genes. Focusing on seed development, we found that many key developmental regulators of seed/endosperm development, including *AGL61*, *AGL62*, *LEC1*, *LEC2*, *ABI3*, and *WRI1*, were located within DMVs. ChIP-seq for five histone modifications in leaves and seeds clearly showed that the vast majority of histone modification peaks were enriched within DMVs, and their remodeling within DMVs has a critical role in the regulation of seed-specific gene expression. Importantly, further experiment analysis revealed that distal DMVs may act as cis-regulatory elements, like enhancers, to activate downstream gene expression.

**Conclusions:**

Our results point to the importance of DMVs and special distal DMVs behaving like enhancers, in the regulation of seed-specific genes, via the reprogramming of histone modifications within DMVs. Furthermore, these results provide a comprehensive understanding of the epigenetic regulator roles in seed development in castor bean and other important crops.

**Supplementary Information:**

The online version contains supplementary material available at 10.1186/s12915-022-01259-6.

## Background

Seeds not only store the genetic information within the embryo for the next generation, but also contain storage materials such as proteins, lipids, and carbohydrates to fuel seed germination and early seedling development. These storage compounds are a vital source of food, feed, and fuel for humans [1]. There is therefore a pressing need to understand the molecular mechanisms controlling seed growth and development, in order to further improve seed quality and size. In flowering plants, seed development begins with double fertilization, which initiates the development of both the embryo and endosperm, which are surrounded by maternal integuments [2]. Seed development represents unique developmental characteristics that are markedly different from other stages of the plant life cycle due to its complex sequence of events and dramatic transcriptional and epigenetic reprogramming [1, 3]. Over the past two decades, the molecular mechanisms of seed development have been deciphered in *Arabidopsis* (*Arabidopsis thaliana*) and crops, leading to the identification of several key master regulators controlling seed development, storage material accumulation, and seed maturation, such as LEAFY COTYLEDON 1 (LEC1), LEC2, FUSCA 3 (FUS3), and ABSCISIC ACID INSENTITIVE 3 (ABI3) [1, 4, 5]. Recent progress in the high-throughput genome and transcriptome sequencing has enabled a rapid identification of seed-specific or seed-stage-specific genes in diverse plant species [6–11], revealing that their precise expression in seeds or at specific stages is critical for proper seed growth and development. Nevertheless, the molecular mechanisms behind the temporal and spatial regulation of these master regulators of seed development are less clear.

Epigenetic mechanisms, such as DNA methylation and histone modifications, have been demonstrated to play critical roles in the regulation of transcriptional reprogramming during plant development [12–14]. In particular, dramatic epigenetic reprogramming takes place during the transition from vegetative to reproductive growth [3, 15, 16]. DNA methylation represents a major epigenetic mark that imposes transcriptional repression onto transposable elements (TEs) and regulates global gene expression [17]. In plants, DNA methylation can occur in three distinct sequence contexts: CG, CHG, and CHH (H=C, A, or T), which are maintained via different and dedicated DNA methyltransferases, for example METHYLTRANSFERASE 1 (MET1) for CG [18], CHROMOMETHYLASE 3 (CMT3) for CHG [19], and CMT2 and DOMAINS REARRANGED METHYLASE1/2 (DRM1/2) for CHH [20]. Genetic analyses with loss-of-function alleles in these methylases have illuminated that they are essential for embryogenesis and seed development [21, 22]. Global profiling of methylation has revealed significant variation in DNA methylation among eukaryotic genomes, but also highlighted several conserved properties such as gene body methylation (gbM) and extensive methylation of transposons [17, 23, 24]. Notably, recent studies in vertebrates and plants have uncovered another unique characteristic of DNA methylation: DNA methylation valleys (DMVs) or unmethylated regions (UMRs), within which DNA methylation levels are less than 5% in all three cytosine contexts [25–30]. DMVs appear to be highly conserved during development and strongly associated with key developmental genes [28, 29]. Usually, DMVs are marked by specific histone marks such as histone H3 trimethylation at lysine 4 (H3K4me3, an active chromatin mark) and H3 trimethylation at lysine 27 (H3K27me3, a repressive mark), to regulate nearby key developmental genes [28–30]. These findings suggest that DMVs are indicative of the regulatory potential of key developmental regulators in appropriate tissues. However, the underlying evolutionary and biological processes that maintain DMVs during development remain unclear in plants. Comparison of DMVs across different plant species will allow the identification of conserved DMVs and will help elucidate their potential functions.

Castor bean (*Ricinus communis*), one member of the Euphorbiaceae family, is often considered a model plant for studies focusing on seed biology in dicots [31]. Unlike most dicotyledonous plants such as Arabidopsis and soybean (*Glycine max*), castor bean seeds have relatively large and persistent endosperm throughout seed development [32, 33], representing substantial structural differences in mature seeds. Castor bean is a major non-edible oilseed crop, whose seed oil is rich in ricinoleic acid (over 90% of total lipids) and is widely used in industry for lubricants, cosmetics, coatings, inks, plastics, and biodiesel [34]. Much progress has been made in understanding the morphological, physiological, and metabolic series of events taking place during seed development in castor bean [31, 32, 35]. Recently, genome and transcriptome sequencing have expanded our understanding of seed development and identified several developmental regulators in castor bean [36–41]. Specifically, the genes involved in ricinoleic acid biosynthesis and glycerolipid assembly have been identified and characterized in castor bean seeds, such as oleate 12-hydroxylase-encoding *FATTY ACID HYDROXYLASE 12* (*FAH12*) [42–44], *DIACYLGLYCEROL ACYLTRANSFERASE 2* (*DGAT2*) [45], *PHOSPHOLIPID:DIACYLGLYCEROL ACYLTRANSFERASE* (*PDAT*) [46], and *PHOSPHOLIPASE D* (*PLDζ2*) [47]. A few regulators of seed development and oil accumulation have been functionally characterized, such as the transcription factors WRINKLED 1 (WRI1) [48] and LEC2 [49]. We showed previously that epigenetic factors, including DNA methylation and genomic imprinting, also regulate seed development in castor bean [33, 50]. In particular, we revealed that DNA hypomethylated regions in castor bean endosperm that behave similarly to DMVs markedly promoted endosperm-specific gene transcription [50]. However, the regulatory genes controlling seed/endosperm development and the accumulation of storage compounds are largely unknown in castor bean, and an integrated comparative transcriptomic and epigenomic analysis has not been attempted.

Here, we first generated a comprehensive gene expression atlas in castor bean by transcriptome deep sequencing (RNA-seq) of 16 diverse tissues and identified seed-specific and seed-stage-specific genes, including those encoding master transcription factors (TFs). We then characterized DMVs in five diverse tissues, including developing seeds, by whole-genome bisulfite sequencing. We discovered that DMVs are highly hypomethylated over development and conserved across divergent plant species. A majority of key seed development regulators are located within DMVs. Notably, we further demonstrated that distal DMVs may act as cis-regulatory elements, like enhancers, to activate transcription. Histone modifications H3K4me3, histone H3 trimethylation at lysine 36 (H3K36me3), histone H3 acetylation at lysine 9 (H3K9ac), histone H3 acetylation at lysine 27 (H3K27ac), and H3K27me3 were significantly enriched within DMVs, and the activity of seed-specific genes was closely correlated with the reconfiguration of histone modifications. Our results provide a comprehensive understanding of the genetic and epigenetic mechanisms underlying seed development in castor bean, highlighting the importance of DMVs as hotspots of regulatory regions for key developmental genes via epigenetic reprogramming. These results will serve as a resource for the precise improvement and molecular breeding of this important oilseed crop or other crops.

## Results

### Global gene expression analyses identify many seed-specific genes

**Fig. 1:**
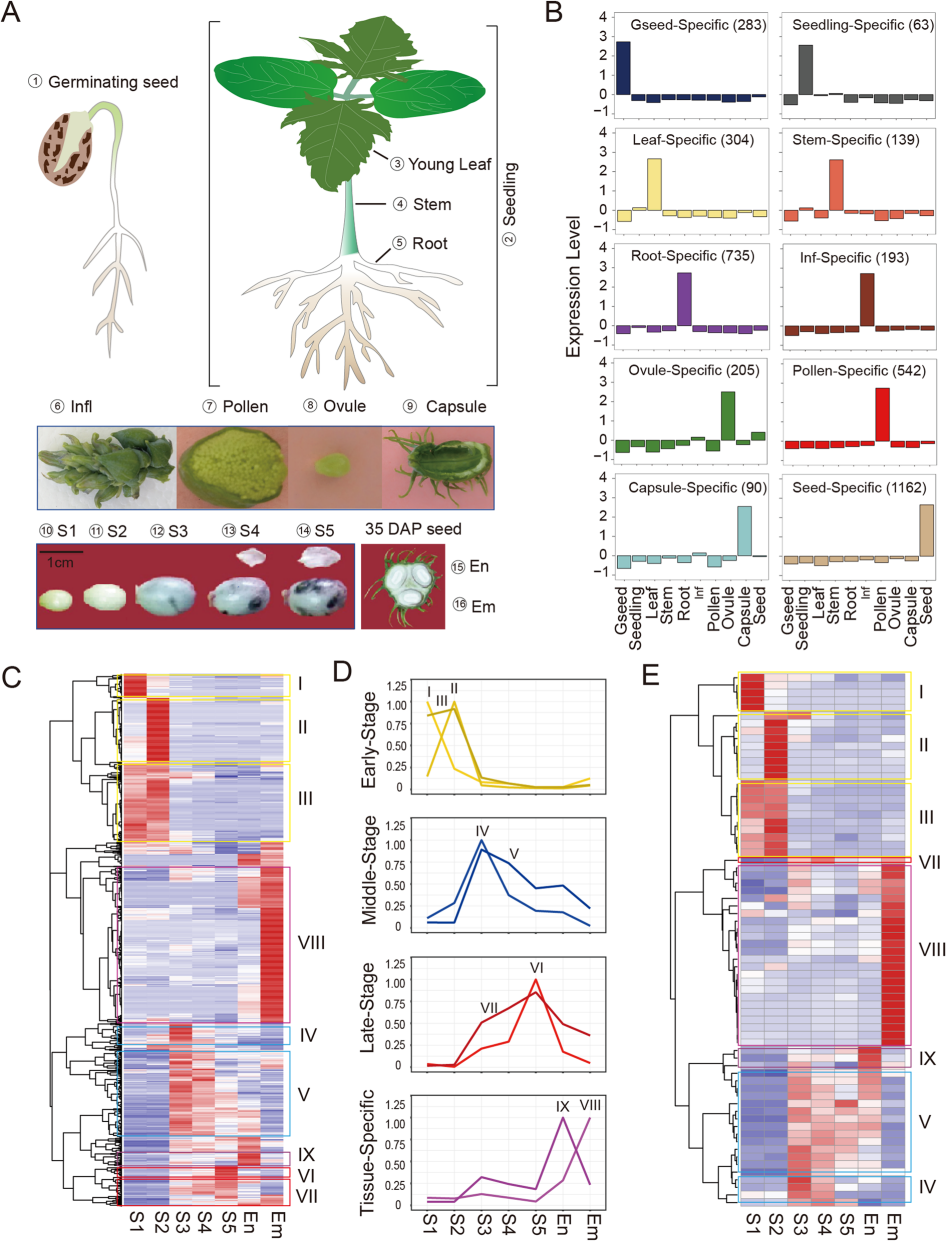
Overview of collected castor bean samples and transcriptome sequencing. **A** List of the 16 samples used for transcriptome deep sequencing. Samples S1–S5 represent seeds at five developmental stages, as described previously by Zhang et al. [35]. DAP, days after pollination. Inf, inflorescence. En, endosperm. Em, embryo. **B** Typical profiles of relative expression levels (as *Z*-score values) of tissue-specific genes across all tissues. The number of genes belonging to each group is shown above the graph. Gseed, germinating seed. **C** Heatmap representation of transcript levels of seed-specific genes throughout seed development. The numbers (from I to IX) denote the clusters whose genes exhibited similar expression profiles. **D** Mean expression of genes within each cluster after normalization (see the “Methods” section). **E** Heatmap representation of transcript levels of seed stage-specific genes encoding transcription factors (TFs) throughout seed development

We first generated a comprehensive gene expression atlas in castor bean based on transcriptome sequencing (RNA-seq) of 16 diverse tissues encompassing the plant entire life cycle (Fig. 1A). RNA-seq yielded ~800.3 million paired-end reads from 16 diverse tissues, and an average, 94.4% of clean reads were mapped to the castor bean genome (Additional file 1: Table S1). We identify, in total, 19,905 genes with an expression level of at least 0.5 FPKM (fragments per kilobase of transcript per million fragments sequenced) in at least one sample (Additional file 1: Table S2). Most samples also showed that many genes expressed at medium levels (5 ≤ FPKM < 10) or high levels (10 ≤ FPKM < 100), with the exception of endosperm and late seed developmental stages (S4 and S5) (Additional file 2: Fig. S1A). Subsequently, we employed the SEGtool package [51] to identify tissue-specific genes based on our expression atlas, resulting in 3716 genes that are specifically or highly expressed in a single tissue (Additional file 1: Table S3). Of them, 1162 genes were specially expressed in seeds (Fig. 1B), as illustrated by Shannon entropy (Additional file 2: Fig. S1B, C), and 1041 genes were seed-stage-specific genes including 71 encoding seed-specific TFs (Additional file 1: Table S4). Upon hierarchical clustering analysis, these seed-specific genes were grouped into nine prominent clusters (from I to IX), roughly corresponding to early (I–III, covering the S1 and S2 stages), middle (IV–V, covering the S3 and S4 stages), and late seed development (VI-VII, covering the S5 stage), with two additional clusters (VIII and IX) consisting of genes specifically expressed in the embryo and endosperm, respectively (Fig. 1C, D and Additional file 1: Table S4). A clustering analysis of seed-specific TFs showed a similar pattern associated with stages of seed development (Fig. 1E). Considering the importance of castor bean as a non-edible oilseed crop, we identified 25 seed-specific genes involved in the biosynthesis of fatty acids and triacylglycerols in castor bean (Additional file 1: Table S5). We performed quantitative reverse transcription PCR (qRT-PCR) for 11 selected seed-specific or seed-stage-specific genes and validated their expression profiles across the sampled tissues (Additional file 2: Fig. S2).

Gene ontology (GO) analysis showed that genes expressed specifically at early stages of seed development were mainly involved in the organization and biogenesis of the cell wall (Additional file 2: Fig. S3A). Several genes encoding known transcription factors such as the MADS-box TFs AGAMOUS-LIKE 61 (AGL61, 29693.m002042), AGL62 (29838.m001673 and 29693.m002041), and AGL80 (30128.m008984) were detected at this stage (Additional file 1: Table S4 and Additional file 2: Fig. S3B), which were previously reported as developmental regulators of endosperm cellularization and early seed development in other plants [52–54]. Seed middle-stage genes mainly participated in lipid biosynthesis, such as fatty acid and triglyceride metabolism (Additional file 2: Fig. S3A), consistent with the onset and continued deposition of storage oils as our previous report [35]. We noticed that 24 seed-specific genes involved in the biosynthesis of fatty acids and triacylglycerols were specifically expressed at this stage. In particular, *FAH12* (28035.m000362) encoding oleate 12-hydroxylase is responsible for the biosynthesis of ricinoleic acid [42], initially expressed at seed middle-stage (Additional file 1: Table S4). We also identified many genes encoding well-studied TFs implicated in regulating seed oil deposition during the oil fast accumulation stage. For example, WRI1 (30069.m000440), a member of APETALA 2 (AP2) family of transcription factors, is one of the critical master regulators with a specific role towards regulating seed oil biosynthesis [55]. LEC1 (29629.m001369) belongs to the nuclear transcription factor Y (NF-Y) family and plays a central role in the control of seed development and storage material accumulation [56–59]. We also identified the two B3 TF members LEC2 (30190.m010868) and FUS3 (30131.m006860), which are generally considered master regulators of embryogenesis and oil accumulation in plants (Additional file 2: Fig. S3B) [49, 60, 61]. At the seed late stage, many genes were associated with protein biosynthesis and seed maturation (Additional file 2: Fig. S3A). For example, genes encoding 2S storage albumin, ricin proteins, late embryogenesis abundant proteins (LEAs), and seed dehydrins and maturation protein were specifically expressed at this stage (Additional file 1: Table S4). Notably, we detected another member of the B3 TF family, ABI3 (30204.m001803), as being highly expressed at this stage (Additional file 2: Fig. S3B); ABI3 is thought to play critical roles in the regulation of protein deposition, seed desiccation, and dormancy in flowering plants [62, 63].

Taken together, these results provided vital information to understand the molecular mechanisms underlying castor bean seed development. Particularly, the seed-stage-specific TFs such as MADS-box TFs, LEC1, LEC2, FUS3, ABI3, and WRI1 may represent key master regulators for various aspects of seed/endosperm development.

### DNA methylation valleys are conserved throughout development and across plant species

Recent evidence showed that DMVs are greatly enriched around seed-specific genes, especially for key developmental regulators in soybean and *Arabidopsis* [28]. We reasoned that similar epigenetic features might be present in other seed types, such as seeds with large and persistent endosperm throughout seed development like those of castor bean. Therefore, we determined the DNA methylation landscape in different tissues: leaves, roots, embryos, and early (20 days after pollination [DAP]) and middle (35 DAP) endosperm in castor bean. We initially compared DNA methylation levels between seed-specific genes and constitutively expressed genes and observed a substantially lower DNA methylation level for seed-specific genes in all tissues tested (Additional file 2: Fig. S4), suggesting that seed-specific genes may be associated with DMVs.

**Fig. 2:**
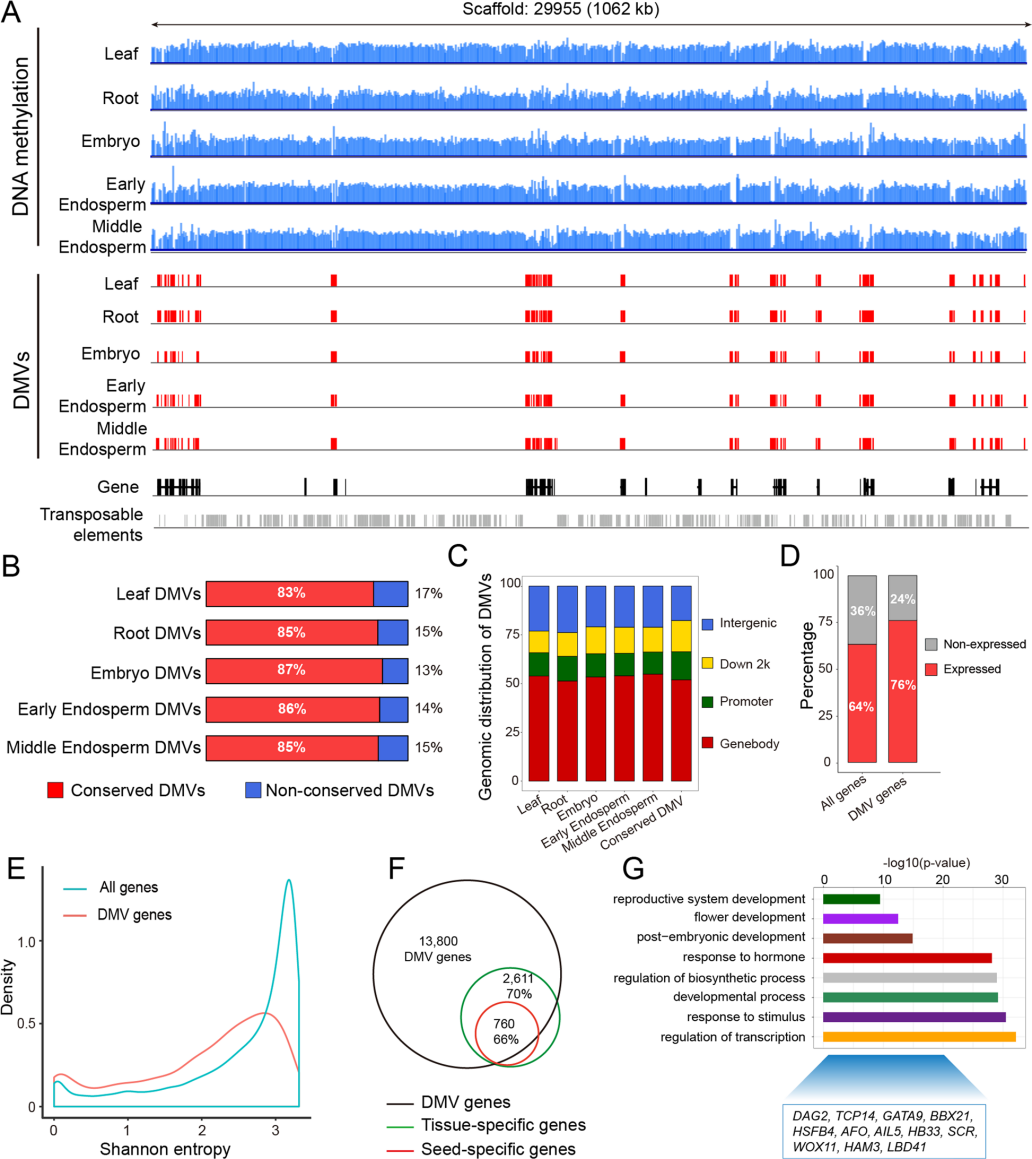
Identification and characterization of DNA methylation valleys (DMVs) in the castor bean genome. **A** Example of DMV distribution along scaffold 29,955 of the castor bean genome. Blue, DNA methylation; red, DMVs; black, genes; gray, transposable elements. **B** Percentage of conserved DMVs that overlap with tissue-specific DMVs. **C** Genomic distribution of DMVs in different tissues. Promoter and down 2k indicate the upstream and downstream 2-kb regions relative to the gene body, respectively. **D** Percentage of expressed and non-expressed genes, genome-wide (all genes) and only within DMVs (DMV genes). **E** Density plot of Shannon entropy values for all genes and DMV genes. **F** Venn diagrams illustrating the overlap between DMVs and tissue-specific or seed-specific genes. **G** Gene ontology enrichment analysis of conserved DMV genes in different plant species (*Arabidopsis*, soybean, and castor bean). Several conserved DMV genes encoding transcription factors are listed below

We then identified castor bean DMVs using the method described by Chen et al. (see the “Methods” section) [28], resulting in 32,567 DMVs shared across different tissues (Additional file 1: Table S6). These DMVs were largely hypomethylated (no more than 2% in all cytosine contexts (Additional file 2: Fig. S5A) and widely distributed in castor bean genome (Fig. 2A), accounting for ~32.8% of genome (115 Mb out of 350 Mb, Chan et al., 2010 [36]). Over 83% of DMVs detected for each tissue was shared with other tissues (Fig. 2A, B), suggesting that DMVs are, to a large extent, conserved across development. Approximately 50% of DMVs overlapped with genes, and another 25% overlapped with flanking regions (within 2 kb on either side) of genes, with the remaining 25% located in intergenic regions named distal DMVs hereafter (2 kb away from the nearest gene) (Fig. 2C). The length distribution of these conserved DMVs was highly consistent with that of genes (Additional file 2: Fig. S5B), indicating that many DMVs are located within gene regions, as mentioned above. We then defined DMV genes as those whose entire gene body and flanking regions (1 kb on either side) were entirely located within DMV regions (see the “Methods” section). A total of 13,800 DMV genes were identified (Additional file 1: Table S7), of which ~76% were expressed in at least one tissue investigated here (Fig. 2D). Shannon entropy analysis showed that most DMV genes are expressed in a very tissue-specific manner (Fig. 2E). For example, ~70.2% (2611 of 3716) of tissue-specific genes were DMV genes, comprising 84.5% of inflorescence-specific genes, 82.7% of root-specific genes, 80.0% of capsule-specific genes, 76.9% of stem-specific genes, 73.8% of pollen-specific genes, 65.4% of seed-specific genes, 60% ovule-specific genes, 59.0% of germinated seed-specific genes and 53.6% of leaf-specific genes (Fig. 2F, Additional file 1: Table S3). And ~81% (196 of 242) tissue-specific TFs and 73.2% (52 of 71) seed-specific TFs were located within DMV regions, suggesting a significant enrichment of TFs within DMVs (*χ*^2^ test, *P*<0.001) (Additional file 2: Fig. S5C). In addition to DMV genes, we identified 250 tissue-specific genes and 89 seed-specific genes associated with DMVs in their promoter regions.

By comparing DMV genes identified in castor bean with those from *Arabidopsis* and soybean, we found that there were 2878 common DMV genes across these three plant species (Additional file 1: Table S8), and developmental genes and genes encoding TFs showed significant enrichment (Fig. 2G). For example, AINTEGUMENTA-LIKE 5 (AIL5), a member of the AP2 family of transcriptional regulators, plays a key role in developmental change from vegetative to embryonic phase [64]. In sum, these results clearly illustrate that DMVs are highly conserved across development and even different plant species and associated with tissue-specific genes.

### Key developmental regulators controlling seed development are present within DMVs

**Fig. 3:**
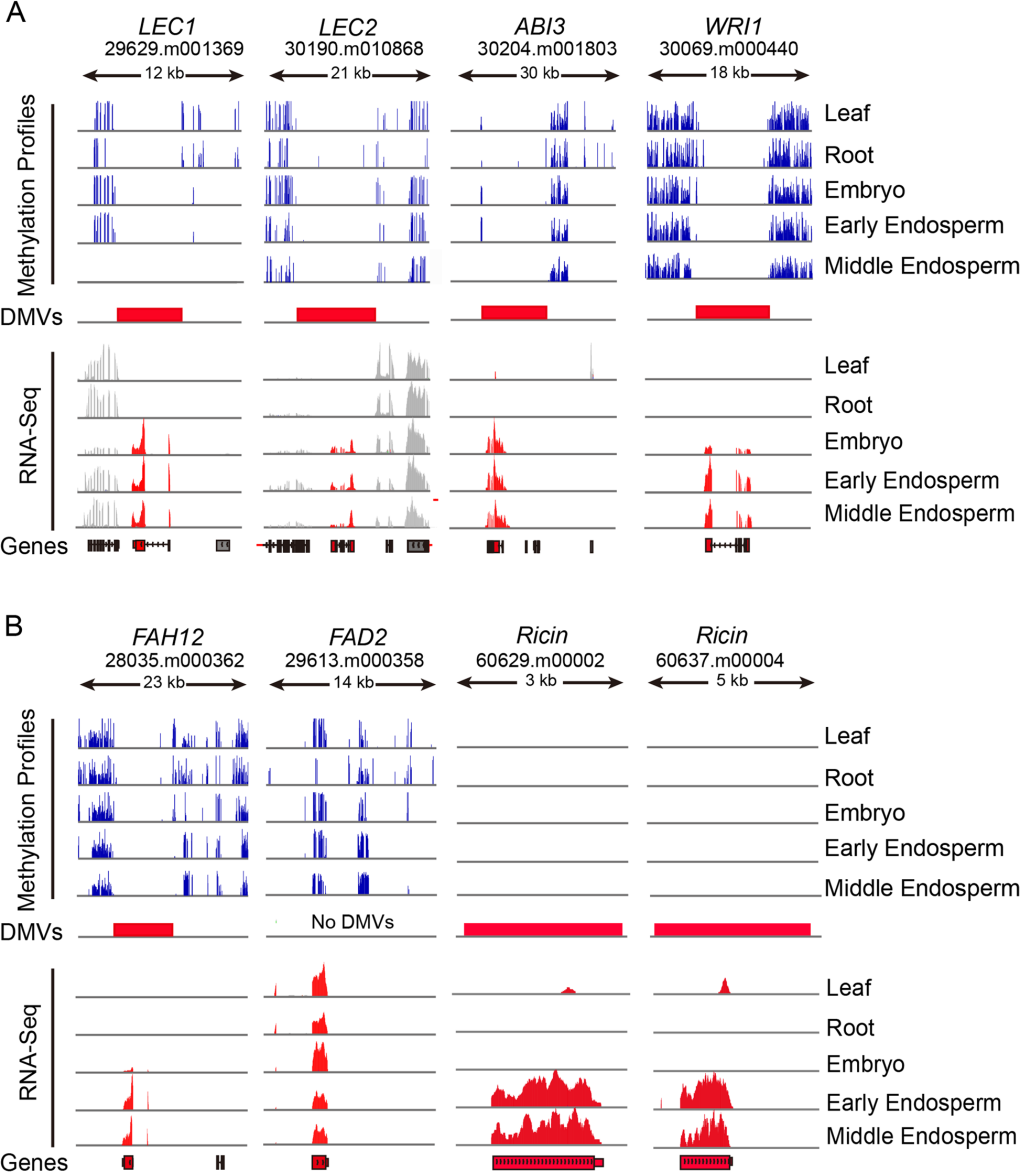
Landscape of genomic DNA methylation and expression profiles for key seed-specific genes across different tissues. **A** Landscape of genomic DNA methylation (Methylation profiles, top) and transcript levels (RNA-seq, bottom) for key seed-specific genes encoding transcription factors (including *LEC1*, *LEC2*, *ABI3*, and *WRI1* indicated by red gene models) across different tissues. **B** Landscape of genomic DNA methylation and transcript levels for key seed-specific genes encoding enzymes (including *FAH12* and *Ricin*s indicated as red gene models) and *FAD2* among different tissues

Focusing on seed development, many seed-specific and seed-stage-specific genes and TFs were preferentially located within DMVs. For example, genes regulating early endosperm development, such as *AGL61* and *AGL62* within DMVs, did not vary significantly with respect to their methylation levels among tissues (Additional file 2: Fig. S6). Several well-studied master regulators governing embryogenesis, oil accumulation, and seed maturation, such as *LEC1*, *LEC2*, *WRI1*, and *ABI3*, also were located within DMVs (Fig. 3A). Also, genes encoding key metabolic enzymes critical for the biosynthesis of ricinoleic acid and ricin were located within DMVs (Fig. 3B). For instance, *FAH12* encoding an oleate hydroxylase enzyme that catalyzes the production of ricinoleic acid (12-OH 18:1Δ^9^) using oleic acid as substrate (18:1Δ^9^) was present within DMVs and specifically expressed at the seed or oil fast accumulation stage. By contrast, its close homolog fatty acid desaturases 2 (*FAD2*, 29613.m000358), encoding a fatty acyl desaturase that converts the same substrate oleic acid (18:1Δ^9^) into linoleic acid (18:2Δ^9,12^), was constitutively expressed and methylated over the length of its gene body (Fig. 3B). We thus hypothesize that divergent gene body methylation between *FAH12* and *FAD2* is associated with their differential expression patterns. In addition, two genes encoding the toxic protein ricin displaying a specific and unique expression in castor bean seeds also located within DMVs and were specifically expressed at seed late stage (Fig. 3B). Together, these results suggest that key genes governing seed development and storage material accumulation in castor bean are preferentially located within DMV regions.

### Histone modifications are substantially enriched in DMVs

**Fig. 4:**
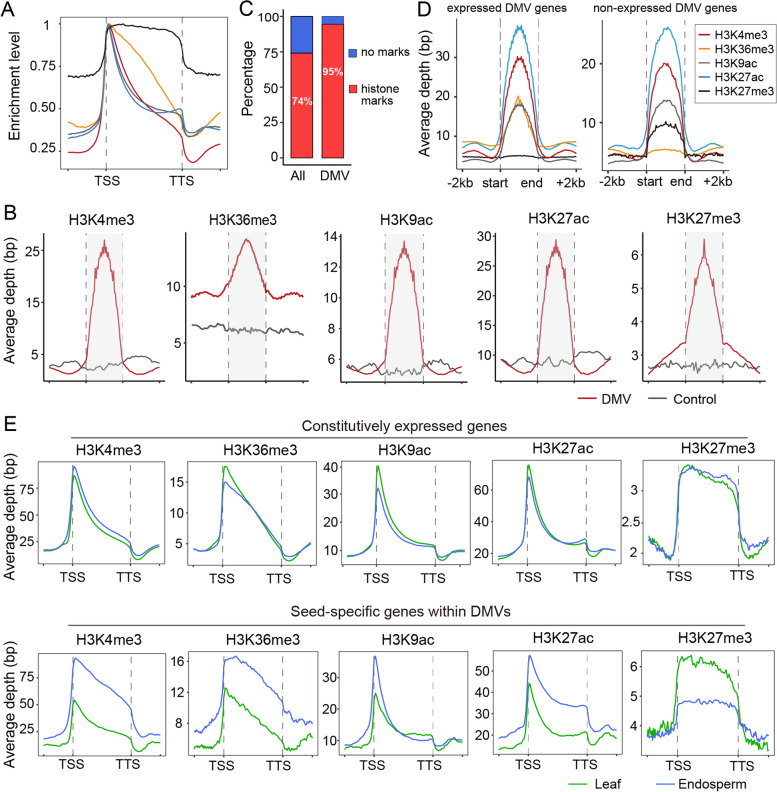
Enrichment analysis of histone modifications within DMVs and DMV genes. **A** Mean enrichment levels of histone modifications around genes (from 2 kb upstream to 2 kb downstream of the gene body). Maximum enrichment level was normalized to 1 for each histone mark. TSS and TTS indicate the transcription start site and transcription termination site, respectively. **B** Average enrichment of histone modifications over DMVs including the flanking 1-kb regions (red lines). The DMV region is indicated by the gray area. Randomized genome regions were used as control regions and shown as gray lines. **C** Proportion of all genes and DMV genes marked by different histone modifications. **D** Mean enrichment levels of different histone modifications around expressed (left panel) and non-expressed DMV genes (right panel) in the endosperm. **E** Average enrichment depth of histone modifications around constitutively expressed genes (up) and seed-specific genes within DMVs (down) in leaves (red lines) and endosperm (cyan lines)

To determine the potential function of DMVs in the regulation of transcription, we profiled the histone modifications genome-wide via chromatin immunoprecipitation followed by sequencing (ChIP-seq). Four active histone marks H3K4me3, H3K36me3, H3K9ac, and H3K27ac and a repressive mark H3K27me3 were investigated in this study. A comparison of biological replicates for each histone mark showed high reproducibility (Additional file 2: Fig. S7A). Consistent with the characteristic features of these histone marks in the genome, all four active histone marks were generally restricted to the transcription start sites (TSS) while repressive mark H3K27me3 was largely enriched over the entire gene body from TSS to TTS (transcription termination sites) (Fig. 4A), thus validating our ChIP experiment and analysis. From the profiling of histone modifications, we identified 23,124 and 21,319 H3K4me3 peaks, 19,448 and 19,989 H3K36me3 peaks, 27,637 and 23,148 H3K9ac peaks, 25,084 and 25,999 H3K27ac peaks, and 9249 and 8168 H3K27me3 peaks in leaf and endosperm tissue, respectively (Additional file 2: Fig. S7B). Over 70% of peaks for H3K4me3, H3K9ac, and H3K27ac marks co-localized, and a relatively small fraction of H3K36me3 peaks overlapped with other active histone marks, as expected (Additional file 2: Fig. S7C). For the H3K27me3 mark, less than 30% of peaks showed corresponding H3K36me3, H3K9ac, or H3K27ac peaks, but ~50% of H3K27me3 peaks had a corresponding H3K4me3 peak, suggesting distinct distribution patterns for active and repressive histone marks across the castor bean genome (Additional file 2: Fig. S7C).

We assessed whether these histone marks were enriched at DMVs. As shown in Fig. 4B, the average enrichment of all histone marks over DMVs was much higher than that of random intergenic regions (control regions). Specifically, we observed that 97% of H3K4me3 peaks, 95% of H3K27ac peaks, 90% of H3K9ac peaks, 87% of H3K36me3 peaks, and 89% of H3K27me3 peaks map within DMVs (Additional file 2: Fig. S7D), which surprisingly covered 95% of DMV genes (Fig. 4C). The active histone marks H3K4me3 and H3K27ac were strikingly enriched over DMV genes, consistent with the fact that most of DMV genes (~76%) are expressed, as mentioned above. Furthermore, the comparison between expressed and non-expressed DMV genes revealed a clear change in the H3K27me3 profile relative to other histone marks, suggesting a potential role for H3K27me3 within DMVs in the regulation of transcription (Fig. 4D). These results showed that these histone marks are strongly associated with DMVs and DMV genes, and/or DMVs have the potential to position histone modifications to regulate transcription.

### Seed developmental regulators are associated with the reconfiguration of histone modifications within DMVs

An outstanding question is how the transcription of these seed-specific genes located within DMVs was specifically activated in seeds or repressed during vegetative growth. Does the reconfiguration of histone modifications within DMVs play a role in the regulation of seed-specific genes during the transition from vegetative growth to seed development? To answer this question, we analyzed the changes in the profiles of different histone modifications between leaf (representing vegetative tissues) and the endosperm (representing the seed). Here we used 35 DAP endosperm instead of whole seeds, as the endosperm (1) represents 95% of the mature seed, (2) is the main site of storage within which many of the key developmental regulators mentioned earlier are highly expressed, and (3) is an atypical example of endosperm persistence in dicots.

We detected 4081 H3K4me3 peaks, 3264 H3K36me3 peaks, 3520 H3K9ac peaks, 5034 H3K27ac, and 2624 H3K27me3 peaks with significant changes between leaf and endosperm samples (Additional file 1: Table S9). Notably, the vast majority of differential peaks (over 80%) occurred within DMVs (Additional file 2: Fig. S7D), covering ~44% (336 of 760) of seed-specific DMV genes (Additional file 1: Table S10). Besides, we noted that there were ~23% (71 of 304) of leaf-specific genes differentially marked by at least one histone modification, suggesting DMVs may be, in a way, associated with the activation of some leaf-specific genes (Additional file 1: Table S11). As illustrated in Fig. 4E, seed-specific DMV genes showed a substantial enrichment for the active histone marks H3K4me3, H3K36me3, H3K9ac, and H3K27ac in the endosperm relative to leaves, and a marked reduction in H3K27me3 levels in the endosperm compared to leaves. By contrast, constitutively expressed genes exhibited no significant differences in their histone modification profiles between the two tissues (Fig. 4E).

**Fig. 5:**
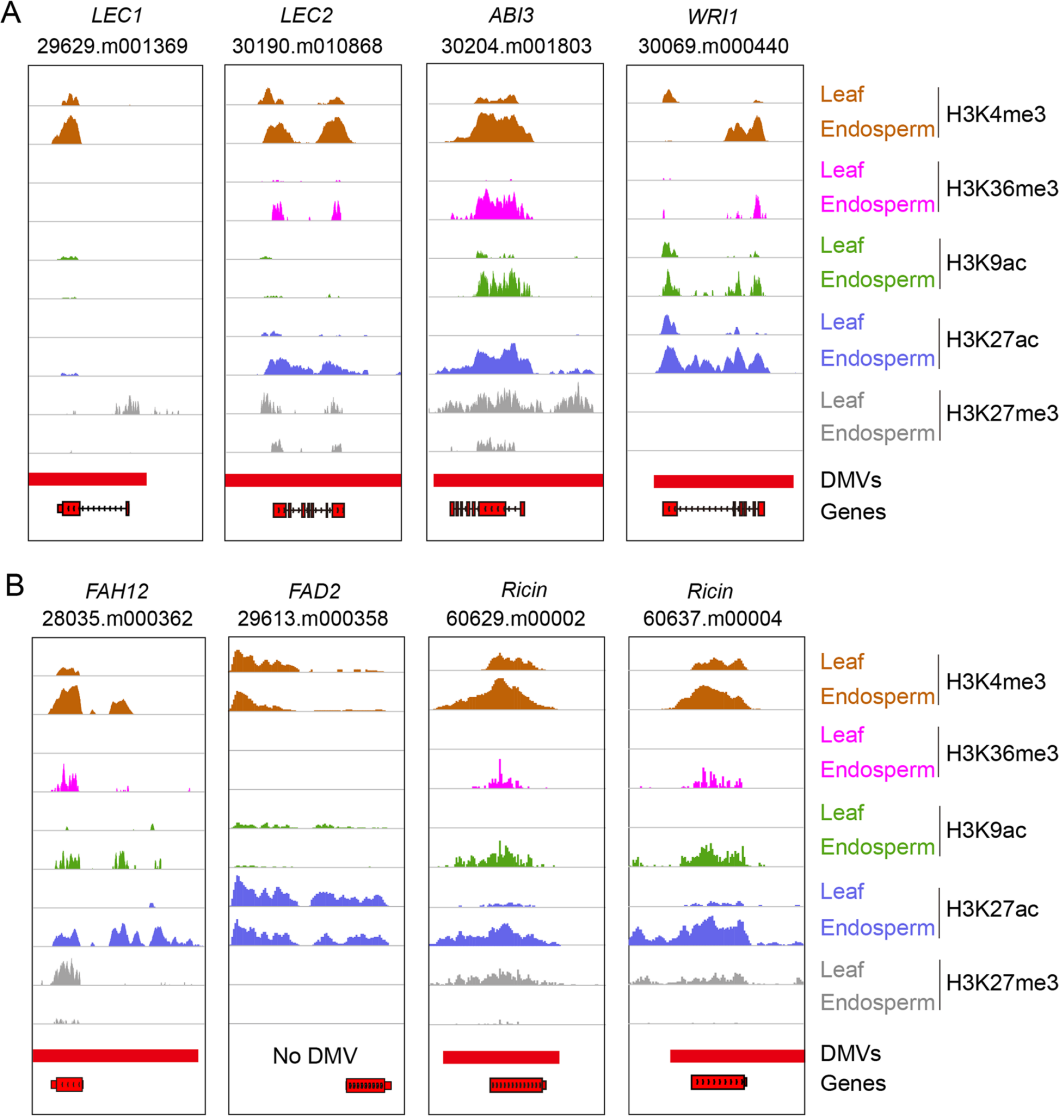
Landscape of histone modifications profiles for key seed-specific genes in leaves and endosperm. **A** Histone modification profiles for key seed-specific genes encoding TFs (including *LEC1*, *LEC2*, *ABI3*, and *WRI1*) in leaves and endosperm. **B** Histone modification profiles for key seed-specific genes encoding enzymes and storage proteins (including *FAH12* and *Ricin*s) and *FAD2* (gene models in red boxes with black outlines) in leaves and endosperm. DMVs are indicated as red horizontal bars

Many important seed-specific DMV genes were potentially regulated by the rearrangement of histone modifications. For example, the master regulators LEC1, LEC2, and ABI3 exhibited a significant enrichment of repressive histone mark H3K27me3 in leaves where they were not expressed, but showed substantially reduced levels of H3K27me3 and higher levels of active marks in seed/endosperm where they were expressed (Fig. 5A). Similarly, *WRI1* showed specific expression in seeds/endosperm, which was closely linked to an increase of active marks at the *WRI1* locus in the endosperm relative to leaves, regardless of H3K27me3 status. In addition, several genes that play critical roles in storage material biosynthesis, such as *FAH12* and *ricin*, displayed similar rearrangements of histone marks in the endosperm relative to leaves, while *FAD2*, a constitutively expressed gene homologous to *FAH12*, experienced no changes for any histone mark (Fig. 5B). ChIP-qPCR performed on multiple tissues (leaves, inflorescences, early seeds, late seeds, endosperm, and germinating seeds) confirmed the changes in the H3K4me3 and H3K27me3 profiles for these key seed-specific genes (Additional file 2: Fig. S8). Overall, our results show that the reconfiguration of histone modifications within DMVs strongly correlates with the transcription potential of seed-specific genes.

### Distal DMVs behave like enhancers

**Fig. 6:**
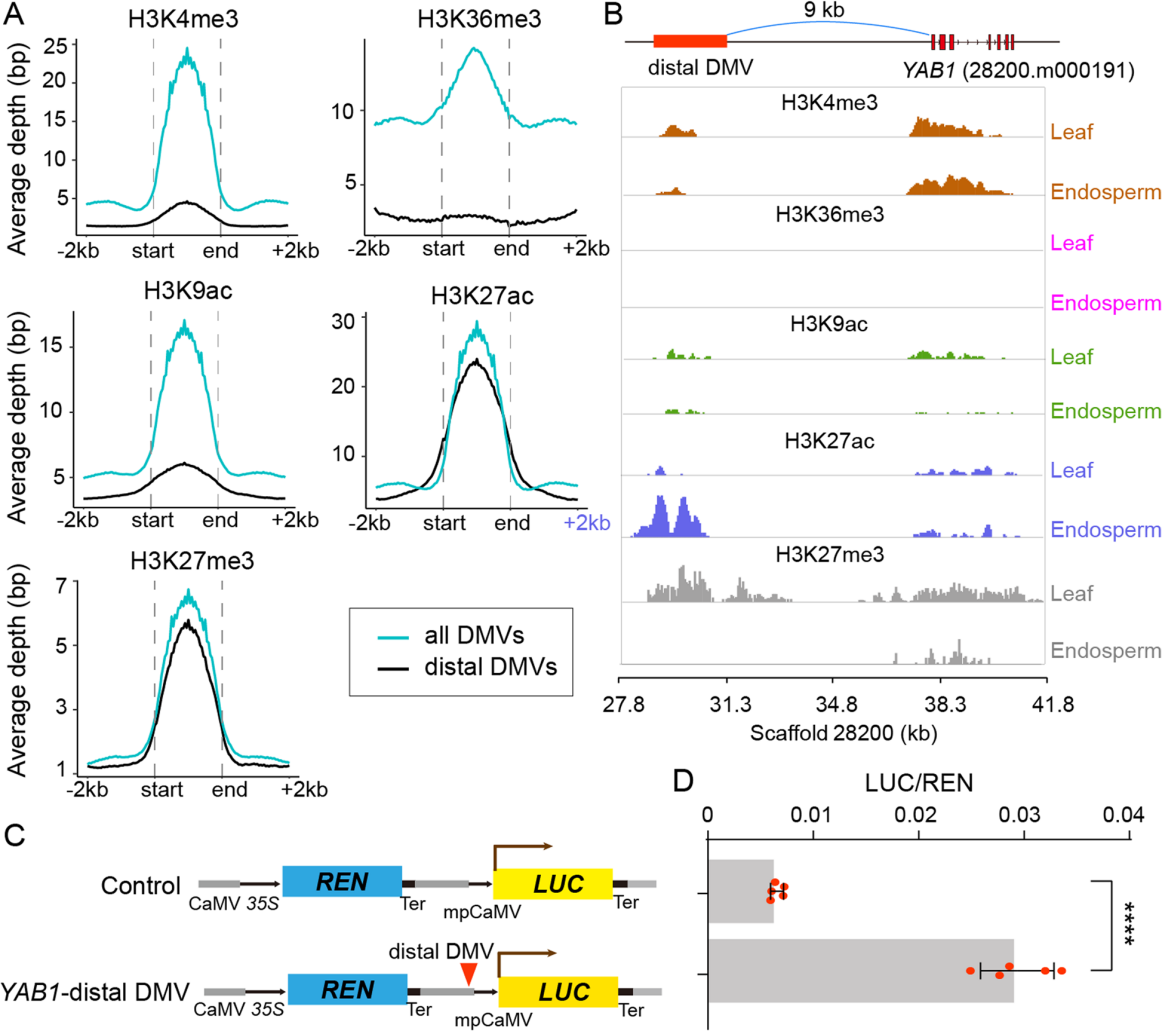
Functional analysis of distal DMVs. **A** Average enrichment of histone modifications over distal DMVs (black lines) and all DMVs (blue lines) in the endosperm. The read count of the ChIP-seq signal for each histone modification was averaged over DMVs and the upstream and downstream 2-kb regions. **B** Example of a distal DMV and its nearest gene *YABBY1* (*YAB1*, upper panel) and landscape of different histone modifications in leaves and endosperm (lower panel). **C** Schematic representation of vectors used for functional validation of the *YAB1*-distal DMV using the dual-luciferase reporter assay system in *Nicotiana benthamiana* protoplasts. **D** Relative luciferase activity, as determined by the ratio of firefly LUC to REN luciferase activity. Error bars indicated SDs from five biological replicates. Statistical significance was determined by a two-sided Student’s *t*-test

In this study, we also identified ~25% (5750) of distal DMVs, defined as being at least 2 kb away from the nearest gene (Additional file 1: Table S12). Using this criterion, we obtained 3566 genes around distal DMVs, of which 168 were seed-specific genes (Additional file 1: Table S12). To determine their potential function, we analyzed the profiles of all histone modifications within all DMVs and distal DMVs. We discovered a striking enrichment of H3K27ac and H3K27me3 marks within distal DMVs, while other histone modifications were strongly depleted from within distal DMVs relative to all DMVs (Fig. 6A). Moreover, for distal DMVs near seed-specific genes, we observed a significant rise in H3K27ac and a reduction of H3K27me3 marks in the endosperm relative to leaves, but no distinct change of other histone modifications (Additional file 2: Fig. S9A). Besides, among 168 seed-specific genes around distal DMVs, there were 45 genes that contained significantly differential peaks of H3K27ac and H3K27me3 between leaf and endosperm in their corresponding distal DMVs (Additional file 1: Table S13).

Considering that active enhancers are generally marked by H3K27ac [65, 66], we speculated that these distal DMVs might act as regulatory cis-acting elements, especially distal enhancers, to regulate the transcription of nearby genes. To test this hypothesis, we selected six distal DMVs (Additional file 1: Table S13 and Additional file 2: Fig. S9B) to validate their potential enhancer activity via a dual-luciferase (LUC) transient transfection assay in *Nicotiana benthamiana* protoplasts. As a result, four distal DMVs showed substantial activation of LUC transcription when driven by a cauliflower mosaic virus (CaMV) 35 promoter and a distal DMV (Additional file 2: Fig. S9B). For example, one distal DMV was located ~9 kb upstream of the seed-specific gene *YABBY1* (*YAB1*, 28200.m000191) (Fig. 6B), a well-characterized gene required for embryo and endosperm development in *Arabidopsis* [67]. ChIP-seq analysis revealed that the active histone modifications H3K3me3, H3K36me3, and H3K9ac display no obvious changes between leaves and the endosperm around *YAB1*, whereas we observed striking changes in H3K27ac and H3K27me3 marks within the distal DMV near *YAB1* (Fig. 6B). Specifically, this distal DMV exhibited a high level of H3K27ac and a low level of H3K27me3 in the endosperm relative to leaves (Fig. 6B), indicating that this distal DMV might be an enhancer region. Dual-LUC transient assay indicated that tobacco protoplasts transfected with the *LUC* reporter gene driven by this distal DMV appear to transcribe *LUC* to a higher level than the control reporter lacking the distal DMV (fold change = 4.5, *P*-value =4.7 × 10^−6^, Fig. 6C, D). Similar changes of histone modifications (Additional file 1: Table S13) and enhancer activity (Additional file 2: Fig. S9B) were also observed in other three distal DMVs of seed-specific genes including *FAH12*, OBL1 (29935.m000048) encoding a seed-specific OIL BODY LIPASE 1 [68], and CLM encoding a CLOMAZONE-RESISTANT PROTEIN involving into the brassinosteroid biosynthesis [69]. These results demonstrated that distal DMVs may act as cis-regulatory elements such as enhancers to activate downstream gene expression.

## Discussion

In this study, we first generated a gene expression atlas covering 16 representative tissues in castor bean, an important non-edible oil crop with a large and persistent endosperm within its seed. Focusing on seed development, we identified 1162 seed-specific genes including 76 encoding TFs that are required for the progression through the seed developmental program. Based on the expression profile of these seed-specific genes, we divided castor bean seed development into three distinct stages: early stage, involving cell division and endosperm cellularization; middle stage, mainly corresponding to embryo development and oil biosynthesis; and late stage, principally involving seed dehydration and accumulation of storage proteins. These three stages are in remarkable agreement with the morphological and metabolic features reported in previous studies [31, 32, 35]. Notably, ~89.5% of seed-specific genes were expressed only at a specific stage during seed development and were largely expressed at the early seed stage—a critical period for seed formation and development, supporting endosperm cellularization and embryogenesis [70]. Several seed-specific TF-encoding genes are known master regulators that govern seed/endosperm development, the accumulation of lipids, and seed maturation, and include *AGL61*, *AGL62*, *AGL80*, *LEC1*, *LEC2*, *FUS3*, *ABI3*, and *WRI1*. We also identified a number of lipid-related genes including *FAH12*, *oleosin1/2*, *DGAT2*, and storage protein-related genes including *ricin*, *2S albumin*, and *LEA*s during castor bean seed development. Besides, it should be noted that most seed-specific TF genes identified in this study have unknown functions but may play critical regulatory roles during seed development as well, and their functions and regulatory network should be determined further.

Recent work in vertebrates and plants has revealed that DMVs are strongly associated with developmental regulators and may behave as regulatory cis-elements [25, 27–30]. Here, we scanned the DNA methylation profiles in diverse tissues and defined 32,567 DMVs across the castor bean genome, representing a significant fraction of the genome. These DMVs are highly stable and conserved across different tissues and developmental stages. Comparison of different plant species indicated a significant variation in the size of the genome represented by DMVs. For example, DMVs covered ~21% of the soybean genome (21,669 DMVs), ~41% of the *Arabidopsis* genome (4,829 DMVs) [28], ~33% of castor bean genome, and ~5.8% of the maize (*Zea mays*) genome (107,583 DMVs) [30]. By contrast, a global survey of DMVs in various vertebrates including mouse (*Mus musculus*) [29] and human [26] only identified ~1000 DMVs, suggesting a significant divergence between animals and plants. Although the number and genome size of DMVs varied significantly, many DMV genes were highly conserved across vertebrates or plant species, suggesting that DMVs may represent a conserved feature among eukaryotic genomes and have an important evolutionary and biological significance. Further study on how DMVs are established and maintained in the genome may provide deeper knowledge into the function of DMVs during evolution and development.

Importantly, we found that the vast majority of DMVs uncovered here were enriched in developmentally important genes, especially genes encoding transcription factor, and strongly associated with tissue-specific genes. For example, several seed-specific DMV genes were located within DMVs and included master developmental regulators (*LEC1*, *LEC2*, *FUS3*, and *ABI3*), fatty acid biosynthesis enzymes (*FAH12*, *Oleosin1/2*, and *DGAT2*) and storage protein genes (*ricin*). Therefore, DMVs have the potential to activate transcription in the appropriate tissues, especially in the case of genes encoding TFs. Intriguingly, examination of the DNA methylation status and expression patterns of *FAH12* and *FAD2*, a pair of homologous genes, showed potential coevolution between gene body methylation and gene expression, as previously reported in cassava (*Manihot esculenta*) [71]. Indeed, DMVs are usually associated with tissue-specific genes, while gene body methylation is strongly associated with constitutively expressed genes. In addition, we noted that some seed-specific genes are not among the DMV genes, but do have DMVs in their promoters (see Fig. 2C). Studies in maize showed that DMVs may act as cis-regulatory elements in promoters and are enriched for TF-binding sites [31]. Accumulating evidence also indicates that DNA–protein interaction sites are generally hypomethylated [72, 73]. If so, one potential scenario would call upon these seed-specific non-DMV genes to be activated by some master TF, itself encoded by a gene present within a DMV, via directly or indirectly binding to their promoter regions, and one worth further inquiry. We also identified many distant DMVs with a strong enrichment for H3K27ac, a characteristic mark of enhancers [65, 66], which suggested that these distal DMVs may act like enhancers to regulate the transcription of the nearest gene. As shown in Fig. S9B, we experimentally confirmed such a regulatory role for several distal DMVs. A similar enhancer effect of DMVs has been reported in maize, where distal unmethylated or hypomethylated regions can regulate downstream gene expression [74]. Distal DMVs might thus represent a novel chromatin signature of plant enhancers for the control of gene expression.

A question central to this study was what drives the expression of seed-specific DMV genes in seeds, or what drives their repression at other stages of development. Histone modifications are thought to contribute to the activation or repression of transcription [75]. Thus, we investigated the profiles of active and repressive histone marks and observed a striking enrichment within DMVs. Significantly, we discovered that many peaks of differential histone marks between leaves and endosperm are also substantially enriched within DMVs, suggesting that DMVs may have important contributions to the positioning and rearrangement of histone modifications during seed formation and development. Seed-specific DMV genes, including master regulators mentioned above, tended to have high levels of active histone modifications (e.g., H3K4me3, H3K36me3, H3K27ac, K3K9ac) and quite low levels of repressive histone modification (H3K27me3) marks in the endosperm relative to leaves, as expected, while constitutively expressed genes exhibited no obvious changes in any of the histone modifications tested here. Overall, our analyses suggest that DMVs are usually marked by histone modifications, and the reconfiguration of histone modifications within DMVs plays a critical role in the regulation of seed-specific genes, especially master developmental regulators.

## Conclusions

We performed an integrated analysis of transcriptome sequencing, whole-genome bisulfite sequencing, and ChIP-seq of histone modifications and provided a comprehensive understanding of the activity of seed-specific genes and the molecular basis of seed/endosperm development in castor bean. In particular, we revealed the crucial role of DMVs in the regulation of key seed regulators in castor bean, especially as cis-acting elements like enhancers. This large dataset will serve as a foundation for understanding the precise regulatory mechanisms underlying seed development in castor bean and other important crops.

## Methods

### Plant materials

The castor bean cultivar “ZB306” (kindly provided by Zibo Academy of Agricultural Sciences, Shandong, China) was used for all experiments. The seeds were surface-sterilized and placed on water-soaked filter papers. After 2 days, the germinated seeds were transferred to soil and seedlings were grown in the greenhouse in 13-h day (28°C)/11-h night (22°C) conditions at Kunming Botanical Garden (Kunming Institute of Botany, Kunming, Yunnan, China). All samples, including germinating seeds (germinated for 2 days), 3-week-old seedlings, young leaves, roots, stems, inflorescence, pollen, ovules, capsules, developing seeds at five stages (10 DAP for S1, 20 DAP for S2, 35 DAP for S3, 45 DAP for S4, 55 DAP for S5), embryos (35 DAP), and endosperm (35 DAP) were collected by manual dissection. The five stages of developing seeds were determined as described previously [35]. The embryo and endosperm samples were separated from seeds at 35 DAP. These samples were immediately frozen in liquid nitrogen and stored at –80°C until total RNA and genomic DNA extraction.

### RNA-seq and data analyses

Total RNA was extracted from different castor bean tissues using Trizol reagent (GENEray, SHH, CHN). The concentration and integrity of the RNA samples were measured on a Qubit 3.0 device (Thermo, Waltham, MA, USA) and by gel electrophoresis (Bio-Rad, Hercules, CA, USA), respectively. A total of 1 μg high-quality RNA was used for library construction with the TruSeq® Stranded mRNA Sample Preparation kit (Illumina, San Diego, CA, USA) following the manufacturer’s instructions. The resulting libraries were sequenced on an Illumina HiSeq X Ten system (Illumina, San Diego, CA, USA) in Shanghai OE Biotech Co., Ltd. (Shanghai, China). After RNA sequencing, raw reads were preprocessed to remove adaptor sequences, low-quality reads, and contaminating sequences. The resulting clean reads were then mapped to the castor bean reference genome (http://oilplants.iflora.cn/) using TopHat [76]. Subsequently, gene expression levels were calculated and normalized as FPKM value using cufflinks [76].

### Identification and characterization of seed-specific genes

Before the identification of tissue-specific genes, genes with FPKM < 2 were omitted in all tested tissues. Then, we employed the ‘SEGtool’ R package [51] to identify tissue-specific genes with the following parameters: SEGtool_result <- SEGtool (expr, exp_cutoff = 2, multi_cpu = 4, detect_mod = 1, result_outdir = ‘SEGtool_result’, draw_heatmap = TRUE, draw_pca = TRUE, draw_plot = TRUE, html_report = TRUE). Gene expression levels were normalized as *Z*-scores, using the following formula: *Z*-score=(*X*_*i*_-*μ*)/*σ*, where *X*_*i*_ is the FPKM value of a gene in tissue *i*, *μ* is the mean FPKM value for the gene across all tissues, *σ* is the standard deviation across all tissues. Next, we calculated Shannon entropy [77] to further estimate the tissue specificity of seed-specific genes. Shannon entropy was calculated using the following formula: *H*(*p*)=-∑*n*!*P*_*i*_log*P*_*i*_, where *P*_*i*_ is the relative gene expression level in tissue *i*. These identified seed-specific genes were subjected to hierarchical cluster analysis using the R package “pheatmap” and to functional enrichment analysis by Kyoto Encyclopedia of Genes and Genomes (KEGG) and GO using the OmicShare online tools (www.omicshare.com/tools).

### Whole-genome bisulfite sequencing (WGBS) and identification of DNA methylation valleys (DMVs)

We performed whole-genome bisulfite sequencing for five diverse tissues: leaves, roots, embryos, and endosperm (35 DAP), all matching RNA-seq samples, as well as an additional sample from early endosperm (20 DAP). Bisulfite sequencing and determination of DNA methylation were conducted as described in our previous studies [33, 50]. Subsequently, DMVs were identified for each sample using the strategy described by Chen et al. [28] with minor modifications. In brief, the entire genome was scanned using a sliding window of 1 kb in 200-bp incremental steps, and only windows containing at least five cytosines with at least fivefold coverage were considered. DNA methylation levels were then calculated for all DNA sequence contexts (CG, CHG, and CHH). DMVs were identified as windows with a methylation level of less than 5% in all three cytosine contexts. Overlapping DMVs were then merged as contiguous DMV regions for further study. DMV genes were identified when the gene and flanking 1-kb regions were located entirely within DMVs.

### ChIP experiments and data analysis

ChIP experiments and analyses were performed according to our previous study [78]. Briefly, young leaves and endosperm (35 DAP, at the fast oil accumulation stage) of castor bean were cross-linked in 1% formaldehyde and the reaction was terminated by adding 0.125 M glycine. Subsequently, the chromatin was extracted from isolated cell nuclei and sonicated to less than 500 bp. Immunoprecipitation was conducted using antibodies specific for five histone modifications: H3K4me3 (07-473; Millipore, Billerica, MA, USA), H3K36me3 (ab9050; abcam, Cambs, UK), H3K9ac (ab32129; abcam, Cambs, UK), H3K27ac (ab177178; abcam, Cambs, UK), and H3K27me3 (a2363; ABclonal, Wuhan, HB, CHN). After immunoprecipitation, genomic DNA was end-repaired, ligated to adapters, and sequenced on a BGISEQ-500 platform (BGI, BJ, CHN).

For ChIP sequencing data, adapter and low-quality reads were trimmed first. Then, the remaining clean reads were mapped to the castor bean reference genome (http://oilplants.iflora.cn/) using bowtie2 (version 2.3.2, [79]). MACS2 software (version 2.2.7) was employed to define peaks in leaf and endosperm samples. Peaks exhibiting differences in binding between leaf and endosperm samples were identified by MAnorm [80]. All assays were performed on two biological replicates.

### qRT-PCR and ChIP-qPCR analysis

Eleven seed-specific genes were selected for RT-qPCR and five genes were selected for ChIP-qPCR analysis in different tissues. The primers for both experiments were designed by Primer 5.0 and listed in Additional file 1: Table S14. For RT-qPCR, total RNA was extracted as mentioned above. First-strand cDNAs were then synthesized with the TransScript All-in-One First-Strand cDNA Synthesis SuperMix for qPCR kit (TransGen, BJ, CHN). qPCR was performed on a Bio-Rad CFX96 system (CA, USA) using TransStart Top Green qPCR SuperMix (TransGen, BJ, CHN). The cycling procedures were as follows: 30 s at 94°C for pre-denaturation; followed by 40 cycles of denaturation (94°C for 5 s) and annealing (60°C for 30 s); a dissociation curve was added after the 40 amplification cycles. For ChIP-qPCR, ChIP-precipitated genomic DNA was used as template for qPCR as described above. Three biological replicates per sample were used for RT-qPCR and ChIP-qPCR. *ACTIN2* was used as reference for normalization.

### Dual-luciferase transient expression assay

To investigate the effect of distal DMVs on gene expression, we performed a dual-LUC transient expression assay in *Nicotiana benthamiana* protoplasts. The pGreen II 0800-Luc vector [81], which harbors a minimal CaMV 35S promoter cloned upstream of the firefly *LUC* reporter gene at the HindIII and BamHI restriction sites, was used as a control vector. Six distal DMV sequences were amplified by PCR and inserted upstream of *LUC* at the KpnI and XhoI restriction sites upstream of the minimal 35S promoter as experimental vectors. In brief, isolated *N. benthamiana* protoplasts [82] were transfected with control or experimental vector, followed by incubation for 16 h at 28°C to allow the transcription of *LUC* and *Renilla* (*REN*) luciferases. *REN* transcription is driven by 35S promoter and provided a control for transfection efficiency. LUC and REN activities were measured with the dual-LUC reporter assay kit (Vazyme, WH, CHN) on a TECAN infinite 200 Microplate reader platform (TECAN, CHE). The activation effect of distal DMVs was then determined as relative LUC activity, normalized to that of REN. For each experiment, at least five biological replicates and eight technical replicates were performed.

## Supplementary Information

**Additional file 1: Table S1.** Summary of RNA-seq data generated from 16 diverse tissues in castor bean. **Table S2.** Genes’ expression level (FPKM) in 16 different tissues of castor bean. **Table S3.** All tissue-specific genes in castor bean. **Table S4.** Seed stage-specific genes in castor bean. **Table S5.** Seed-specific genes related to lipid biosynthesis pathways in castor bean. **Table S6.** Conserved DNA methylation valleys (DMVs) in castor bean. **Table S7.** DMV genes in castor bean. **Table S8.** Conserved DMV genes among Arabidopsis, Soybean and Castor bean. **Table S9.** Differential histone modification regions between leaf and endosperm. **Table S10.** Seed-specific DMV genes potentially regulated by histone modifications. **Table S11.** Leaf-specific DMV genes potentially regulated by histone modifications. **Table S12.** Distal DMVs and the nearest genes in castor bean. **Table S13.** Distal DMVs of seed-specific genes potentially regulated by H3K27ac and/or H3K27me3. **Table S14.** All primers used in this study.

**Additional file 2: Fig. S1.** Gene expression and tissue-specific genes in castor bean. **Fig. S2.** Relative expression level of 11 seed-specific genes in different tissues of castor bean via quantitative reverse transcription PCR (qRT-PCR). **Fig. S3.** Gene ontology (GO) analysis of seed stage-specific genes. **Fig. S4.** DNA methylation level of seed-specific (red line) and constitutively expressed genes (black line) in all investigated tissues. **Fig. S5.** Characterization of DMVs identified in castor bean genome. **Fig. S6.** Landscape of genomic DNA methylation and expression profiles for AGL genes among different tissues. **Fig. S7.** Chip-seq analysis of different histone modifications and their enrichment level around DMVs. **Fig. S8.** ChIP-qPCR analysis of H3K4me3 (up panel) and H3K27me3 (down panel) for key seed DMV genes (including LEC1, LEC2, ABI3, WRI1 and FAH12) in different tissues (root, inflorescence, seed2 (S2), seed4 (S4), endosperm and germinating seed). **Fig. S9.** Changes of histone modifications over those distal DMVs that is near seed-specific genes and experimental validation of distal DMVs as enhancer by the dual-luciferase reporter assay system in *N. benthamiana* protoplasts.

## Data Availability

The RNA-seq, bisulfite-seq, and ChIP-seq data generated in this study have been deposited in NCBI under the BioProject accessions PRJNA787114 (https://www.ncbi.nlm.nih.gov/sra/?term=PRJNA787114), PRJNA787248 (https://www.ncbi.nlm.nih.gov/sra/?term=PRJNA787248), and PRJNA787371 (https://www.ncbi.nlm.nih.gov/sra/?term=PRJNA787371), respectively.

## Abbreviations

DMVs: DNA methylation valleys
LEC1: LEAFY COTYLEDON 1
LEC2: LEAFY COTYLEDON 2
FUS3: FUSCA 3
ABI3: ABSCISIC ACID INSENTITIVE 3
TEs: Transposable elements
MET1: METHYLTRANSFERASE 1
CMT3: CHROMOMETHYLASE 3
CMT2: CHROMOMETHYLASE 2
DRM1: DOMAINS REARRANGED METHYLASE 1
DRM2: DOMAINS REARRANGED METHYLASE 2
gbM: Gene body methylation
UMRs: Unmethylated regions
H3K4me3: Histone H3 trimethylation at lysine 4
H3K27me3: Histone H3 trimethylation at lysine 27
FAH12: FATTY ACID HYDROXYLASE 12
DGAT2: DIACYLGLYCEROL ACYLTRANSFERASE 2
PDAT: PHOSPHOLIPID:DIACYLGLYCEROL ACYLTRANSFERASE
PLDζ2: PHOSPHOLIPASE D
WRI1: WRINKLED 1
RNA-seq: Transcriptome deep sequencing
TFs: Transcription factors
H3K36me3: Histone H3 trimethylation at lysine 36
H3K9ac: Histone H3 acetylation at lysine 9
H3K27ac: Histone H3 acetylation at lysine 27
FPKM: Fragments per kilobase of transcript per million fragments sequenced
qRT-PCR: Quantitative reverse transcription PCR
GO: Gene ontology
AGL61: AGAMOUS-LIKE 61
AGL62: AGAMOUS-LIKE 62
AGL80: AGAMOUS-LIKE 80
AP2: APETALA 2
NF-Y: Nuclear transcription factor Y
LEAs: Late embryogenesis abundant proteins
DAP: Days after pollination
AIL5: AINTEGUMENTA-LIKE 5
FAD2: Fatty acid desaturases 2
ChIP-seq: Chromatin immunoprecipitation followed by sequencing
TSS: Transcription start sites
TTS: Transcription termination sites
LUC: Dual-luciferase
CaMV: Cauliflower mosaic virus
KEGG: Kyoto Encyclopedia of Genes and Genomes
REN: *Renilla*
