# Supplementary material for: Epigenetic regulation of seed-specific gene expression by DNA methylation valleys in castor bean
Source: BMC Biol. 2022 Mar 1;20:57. doi: 10.1186/s12915-022-01259-6 (PMC8886767; doi:10.1186/s12915-022-01259-6)
Supplement: Supplementary file 2 — Additional file 2: Fig. S1. Gene expression and tissue-specific genes in castor bean. Fig. S2. Relative expression level of 11 seed-specific genes in different tissues of castor bean via quantitative reverse transcription PCR (qRT-PCR). Fig. S3. Gene ontology (GO) analysis of seed stage-specific genes. Fig. S4. DNA methylation level of seed-specific (red line) and constitutively expressed genes (black line) in all investigated tissues. Fig. S5. Characterization of DMVs identified in castor bean genome. Fig. S6. Landscape of genomic DNA methylation and expression profiles for AGL genes among different tissues. Fig. S7. Chip-seq analysis of different histone modifications and their enrichment level around DMVs. Fig. S8. ChIP-qPCR analysis of H3K4me3 (up panel) and H3K27me3 (down panel) for key seed DMV genes (including LEC1, LEC2, ABI3, WRI1 and FAH12) in different tissues (root, inflorescence, seed2 (S2), seed4 (S4), endosperm and germinating seed). Fig. S9. Changes of histone modifications over those distal DMVs that is near seed-specific genes and experimental validation of distal DMVs as enhancer by the dual-luciferase reporter assay system in N. benthamiana protoplasts. [file 12915_2022_1259_MOESM2_ESM.docx]

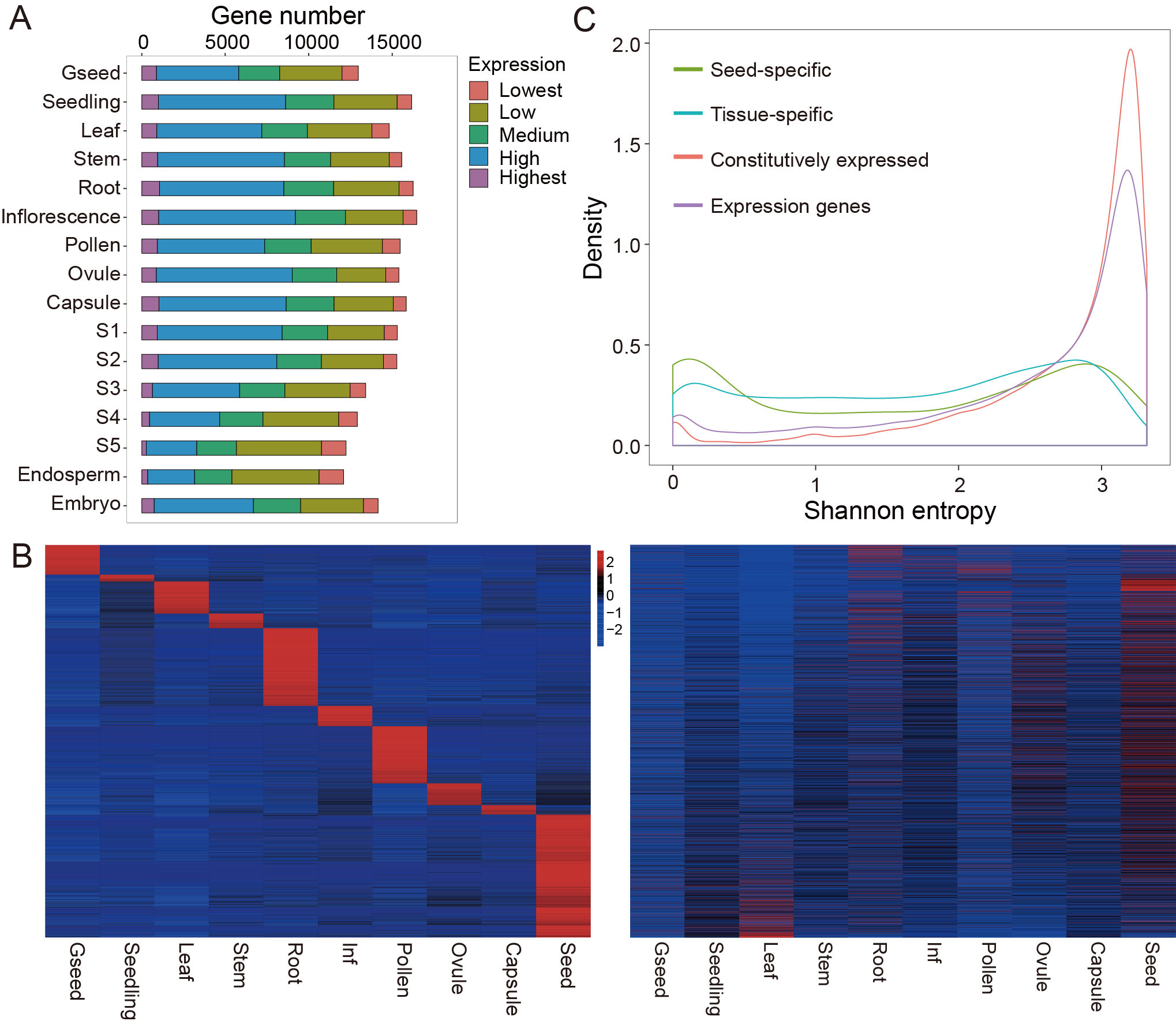


**Fig. S1.** **Gene expression and tissue-specific genes in castor bean.**

1. Numbers of genes with different expression levels in 16 samples. Lowest (0.5 ≤ FPKM < 1); Low (1 ≤ FPKM < 5); Medium (5 ≤ FPKM < 10); High (10 ≤ FPKM < 100); Highest (FPKM ≥ 100). Gseed represents germinating seed.
2. Heatmap represents the expression pattern of tissue-specific genes (left panel) and all expressed genes (right panel) among diverse tissues in castor bean. Gseed and Inf represent germinating seed and inflorescence, respectively. Seed sample includes S1-S5, embryo and endosperm. The color bar indicates the expression level of gene (log_2_ FPKM).
3. Tissue specificity analysis by using Shannon entropy values. The lower entropy value means the higher tissue specificity.

**
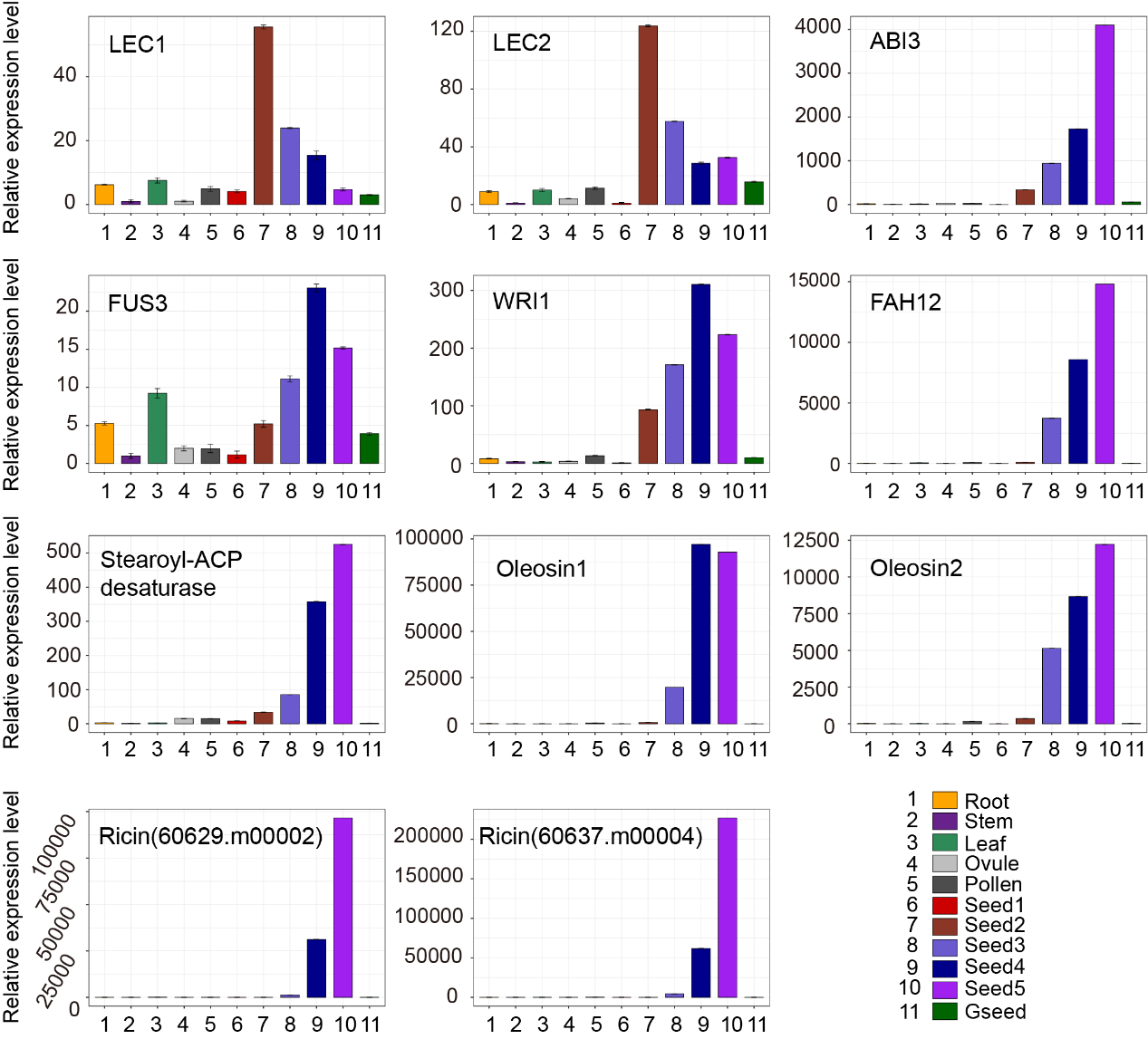
**

**Fig. S2.** Relative expression level of 11 seed-specific genes in different tissues of castor bean via quantitative reverse transcription PCR (qRT-PCR). The error bars show the standard error with three biological replicates. Gseed represents germinating seed. Seed1-Seed5 represent seeds at five development stages.


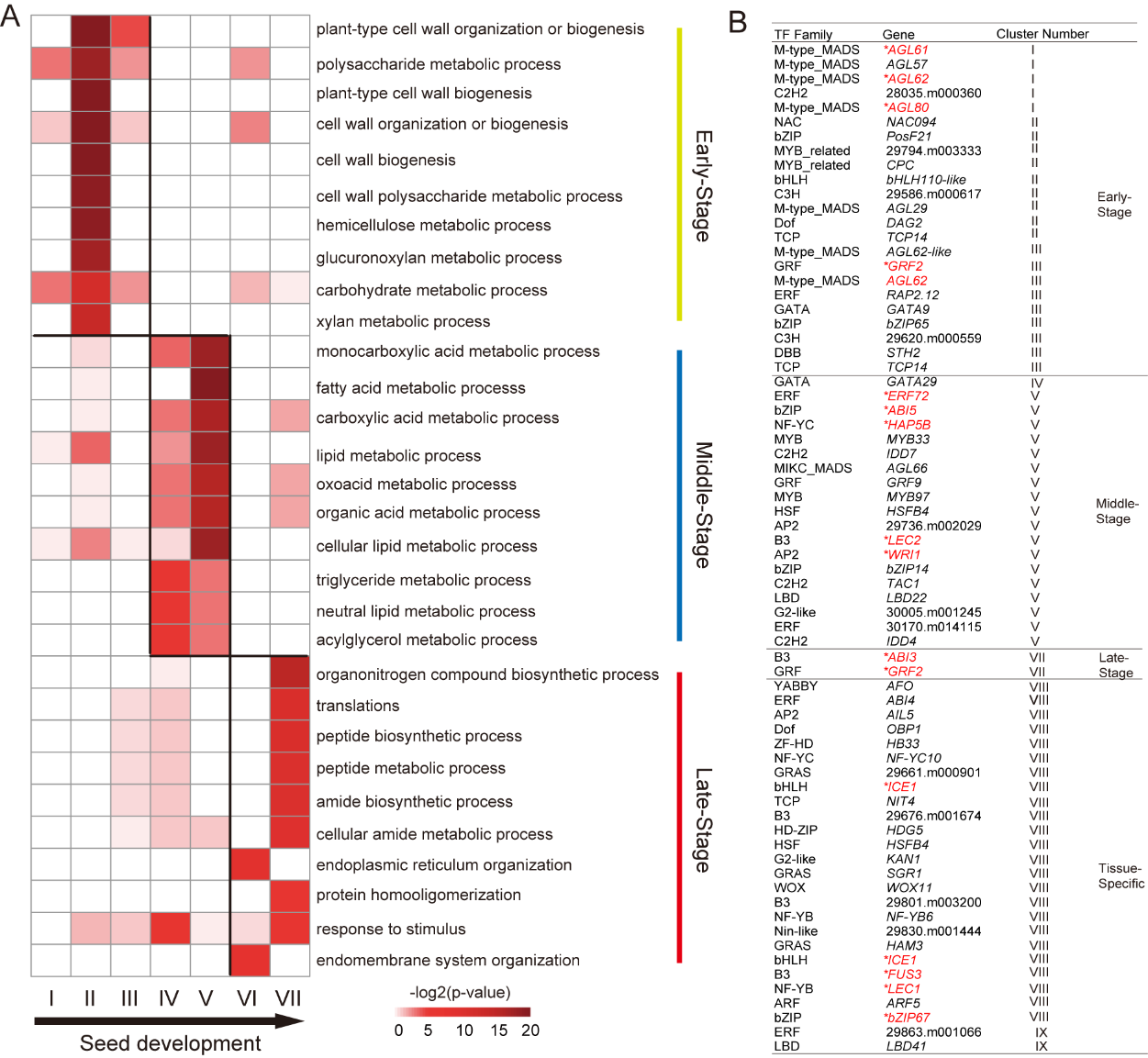


**Fig. S3. Gene ontology (GO) analysis of seed stage-specific genes.**

1. Significantly enriched GO terms among seed stage-specific genes.

**(B)** Stage specificity of seed-specific transcription factors (TF). Asterisks indicate the TFs that are well-studied key regulators of Arabidopsis seed development and storage material accumulation.


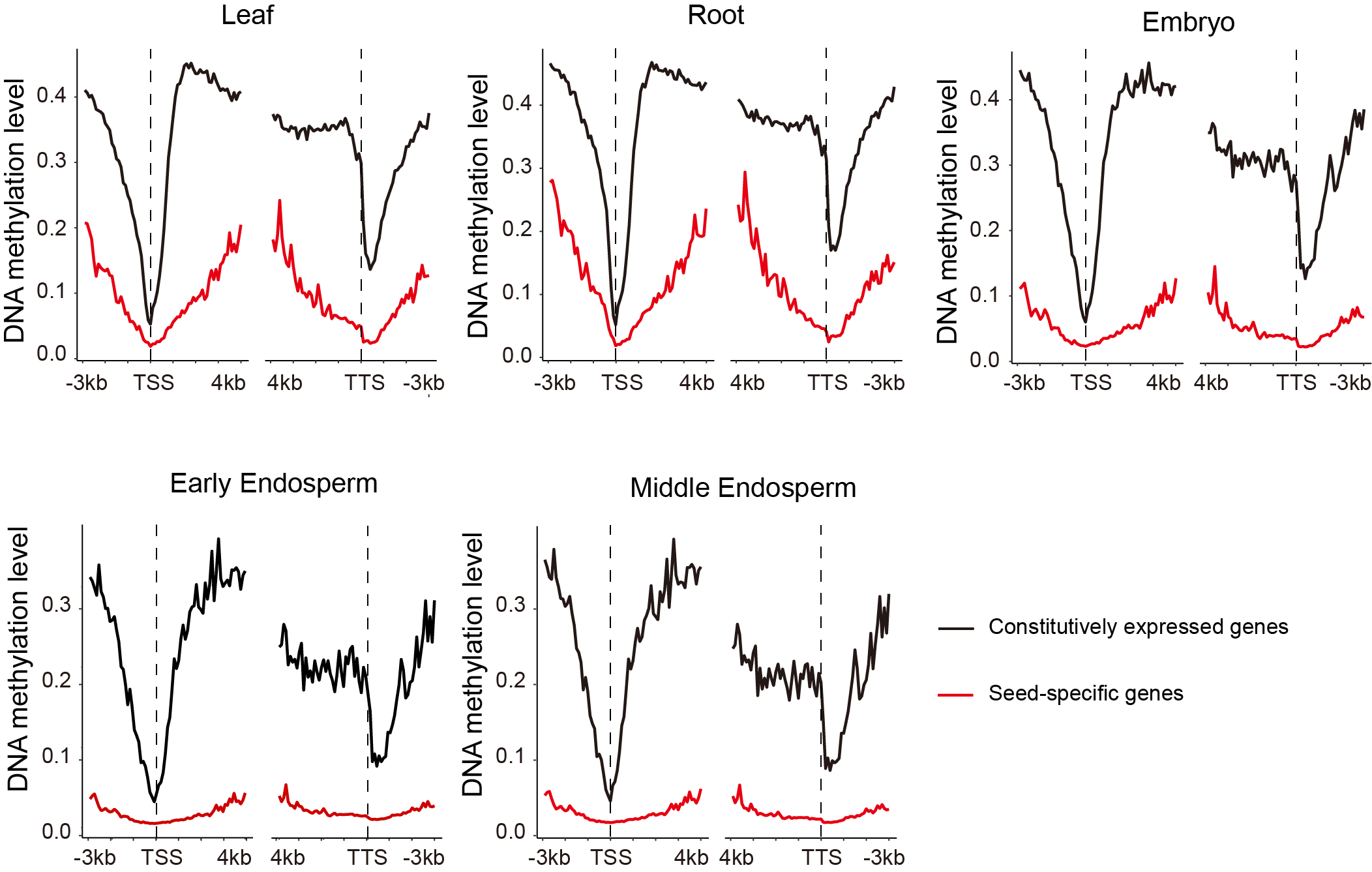


**Fig. S4.** DNA methylation level of seed-specific (red line) and constitutively expressed genes (black line) in all investigated tissues. TSS, transcription start site; TTS, transcription termination site.


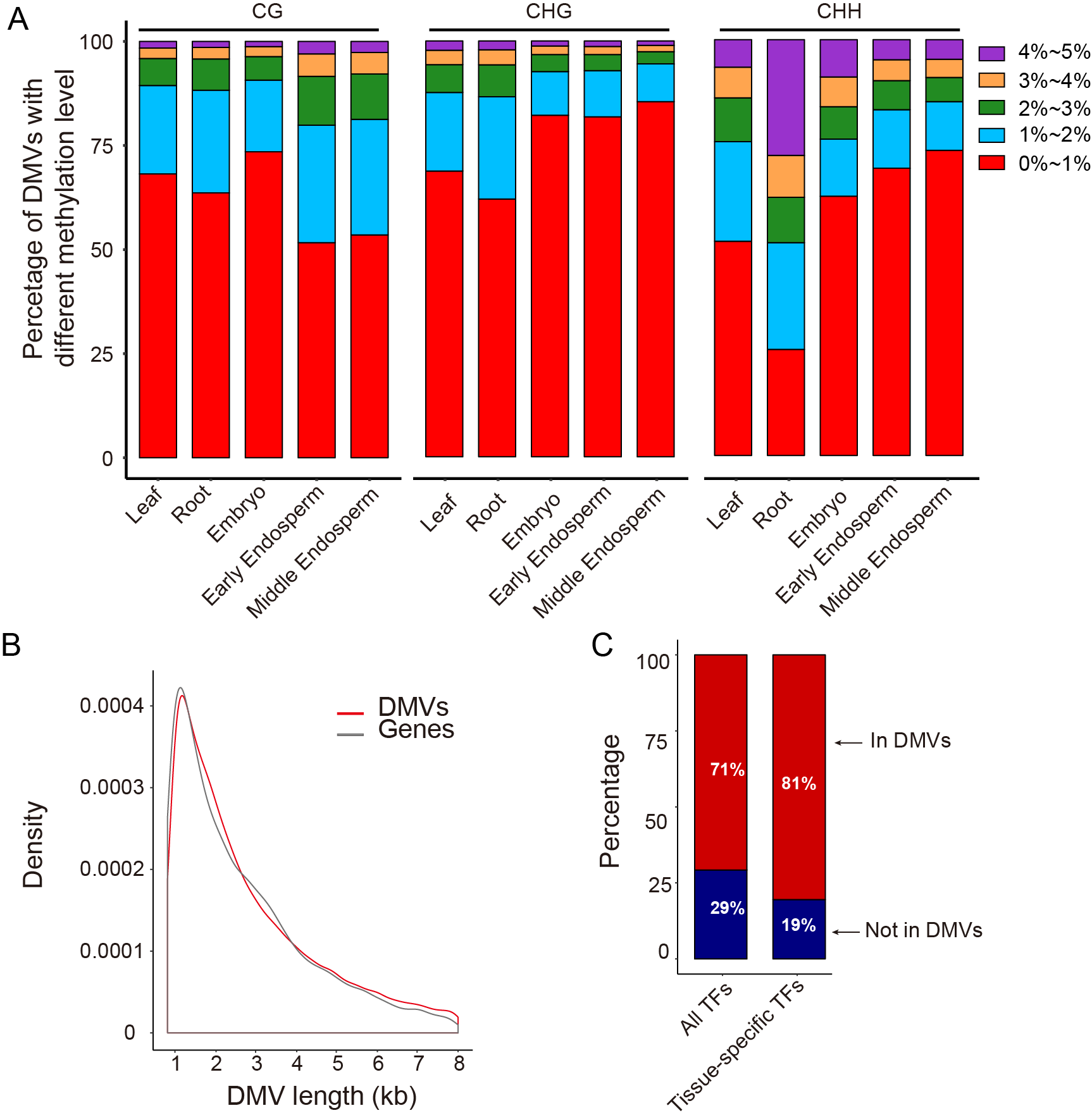


**Fig. S5. Characterization of DMVs identified in castor bean genome.**

1. DNA methylation level of three sequence contexts (CG, CHG, CHH) within DMVs in leaf, root, embryo, early endosperm and middle endosperm.
2. The length of DMVs identified in this study.
3. Comparison of proportion of transcription factors (TFs) between DMV regions and non-DMV regions.


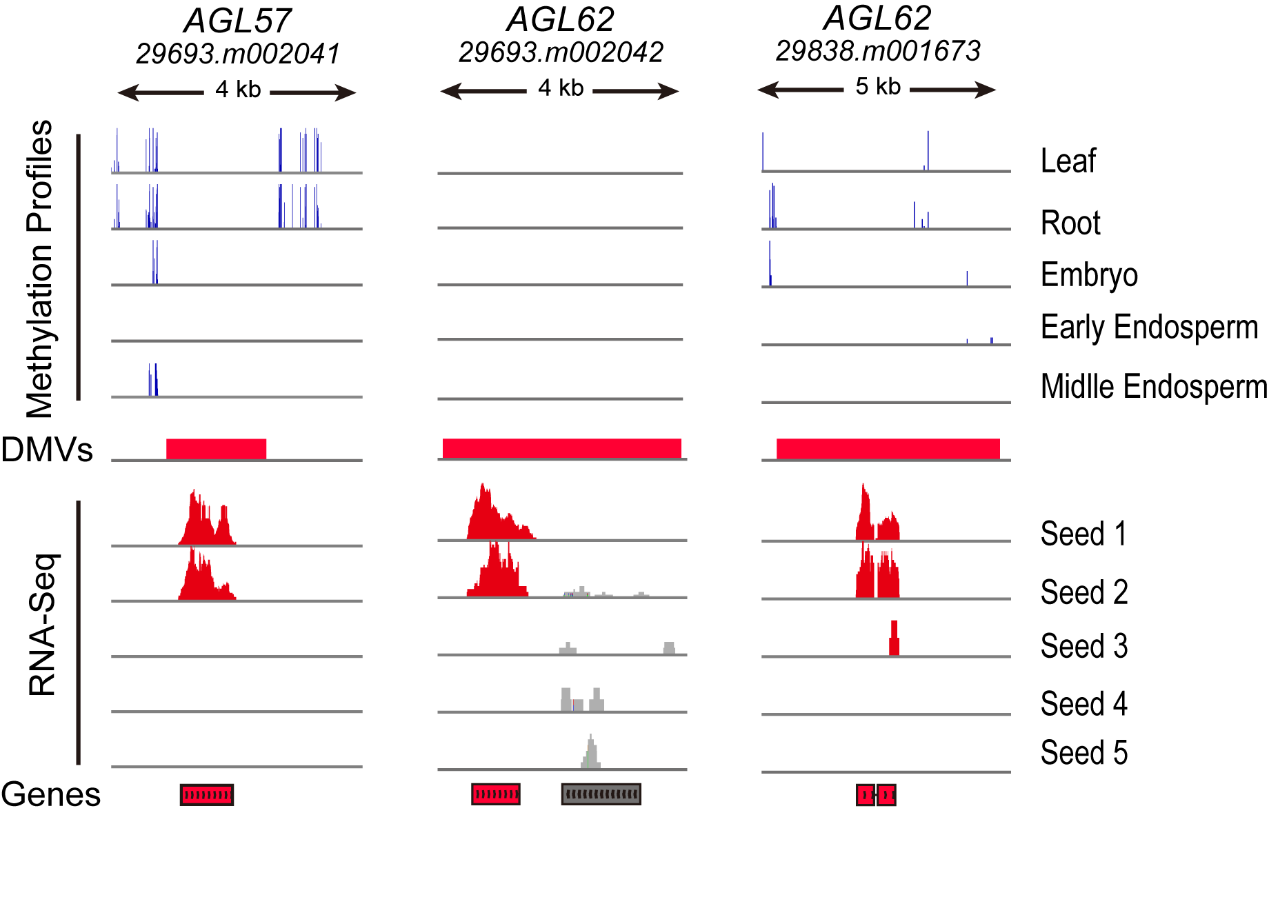


**Fig. S6. Landscape of genomic DNA methylation and expression profiles for *AGL* genes** **among different tissues.**


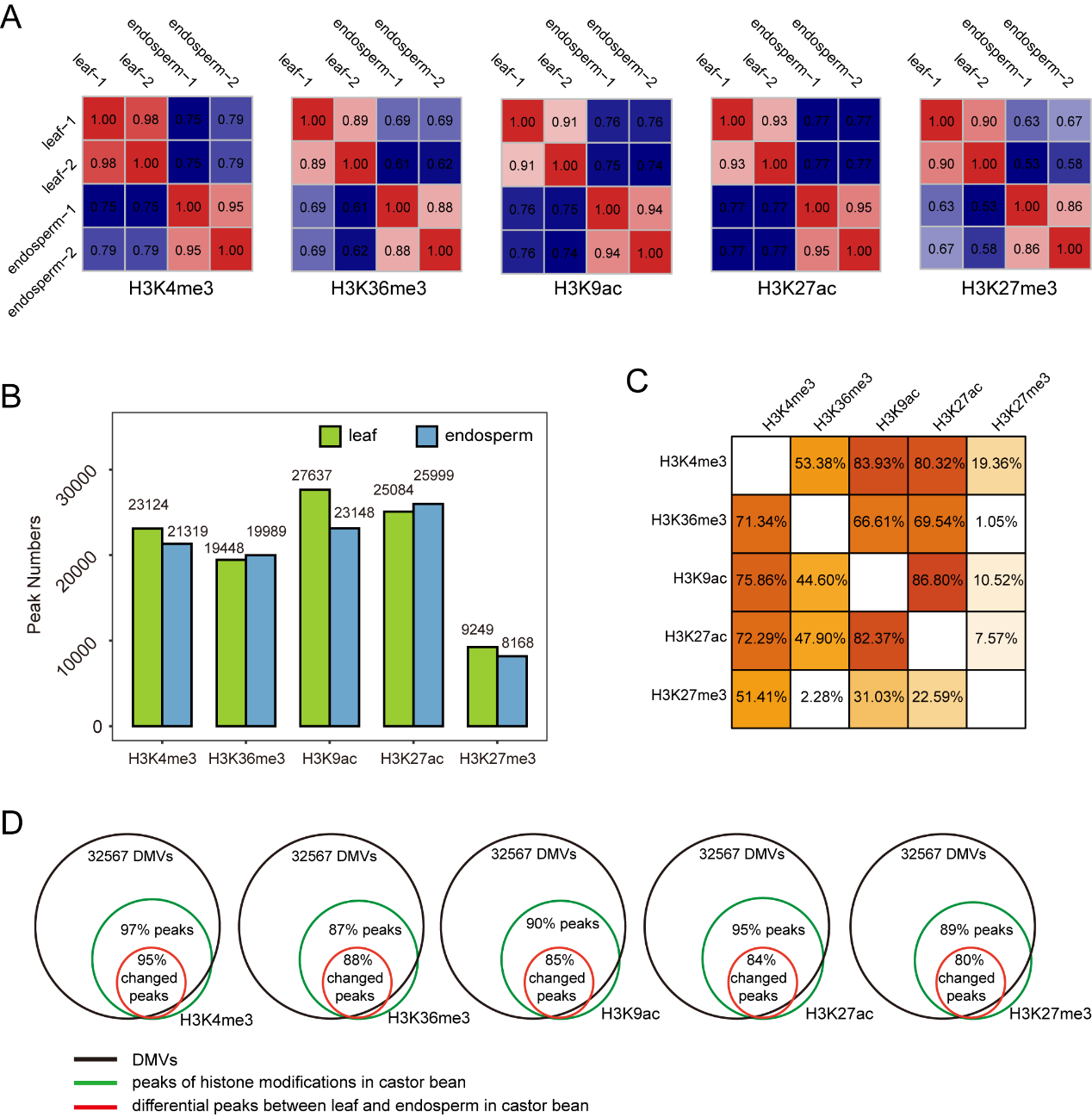


**Fig. S7 Chip-seq analysis of different histone modifications and their enrichment level around DMVs.**

**(A)** The correlation of two biological replicates of different histone marks including H3K4me3, H3K36me3, H3K9ac, H3K27ac and H3K27me3 in leaf and endosperm.

**(B)** The peak number of different histone marks in leaf and endosperm.

**(C)** The percentage of colocalization of any two histone marks. The percentage number indicates the possibility that a histone modification peak on the x-axis exists in a given histone modification peak on the y-axis.

**(D)** Overlap between DMV and peaks in each histone modification. Percentages refer to the percentage of overlapped peaks to total peaks.

**
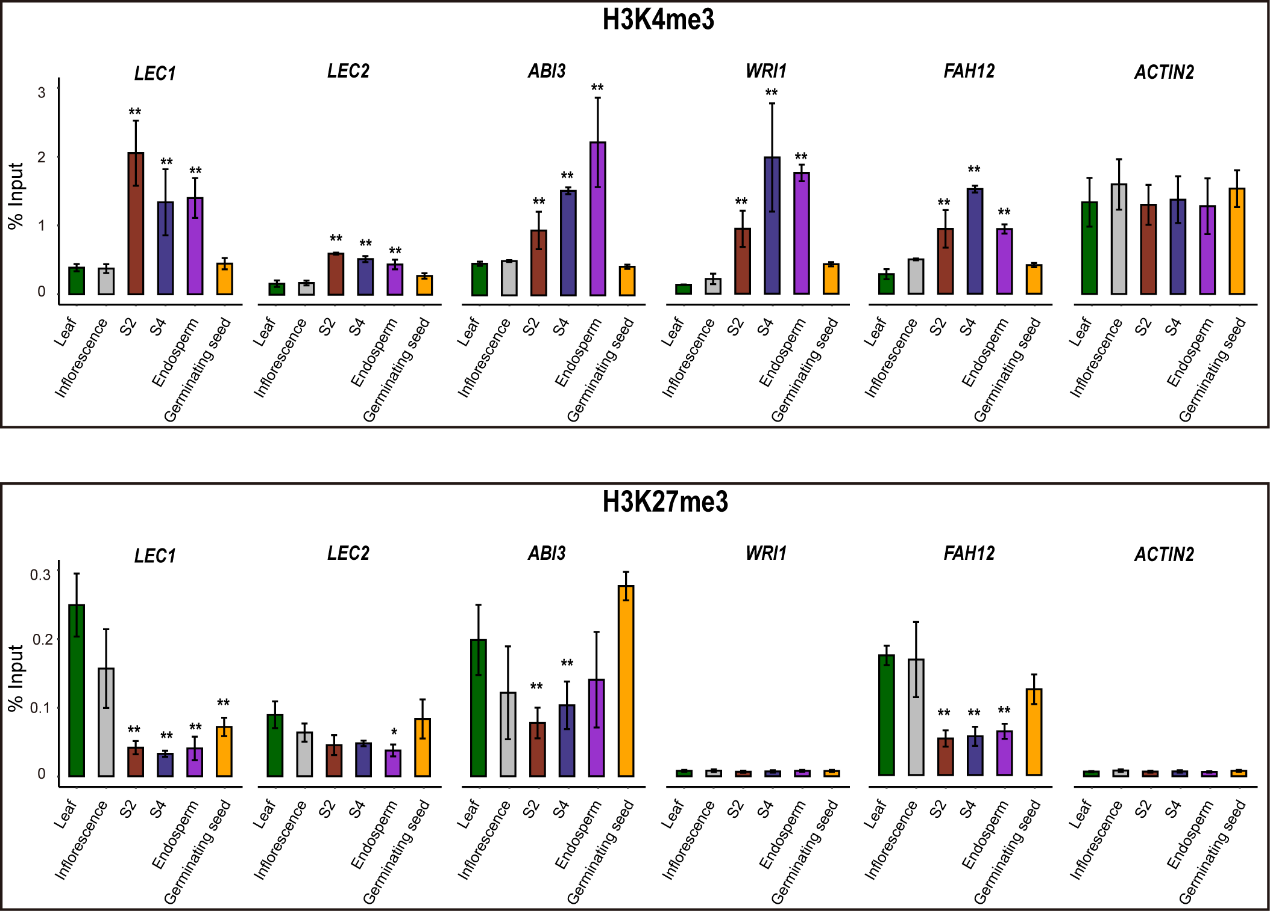
**

**Fig. S8.** ChIP-qPCR analysis of H3K4me3 (up panel) and H3K27me3 (down panel) for key seed DMV genes (including *LEC1*, *LEC2*, *ABI3*, *WRI1* and *FAH12*) in different tissues (root, inflorescence, seed2 (S2), seed4 (S4), endosperm and germinating seed). *ACTIN2* gene was used as control.


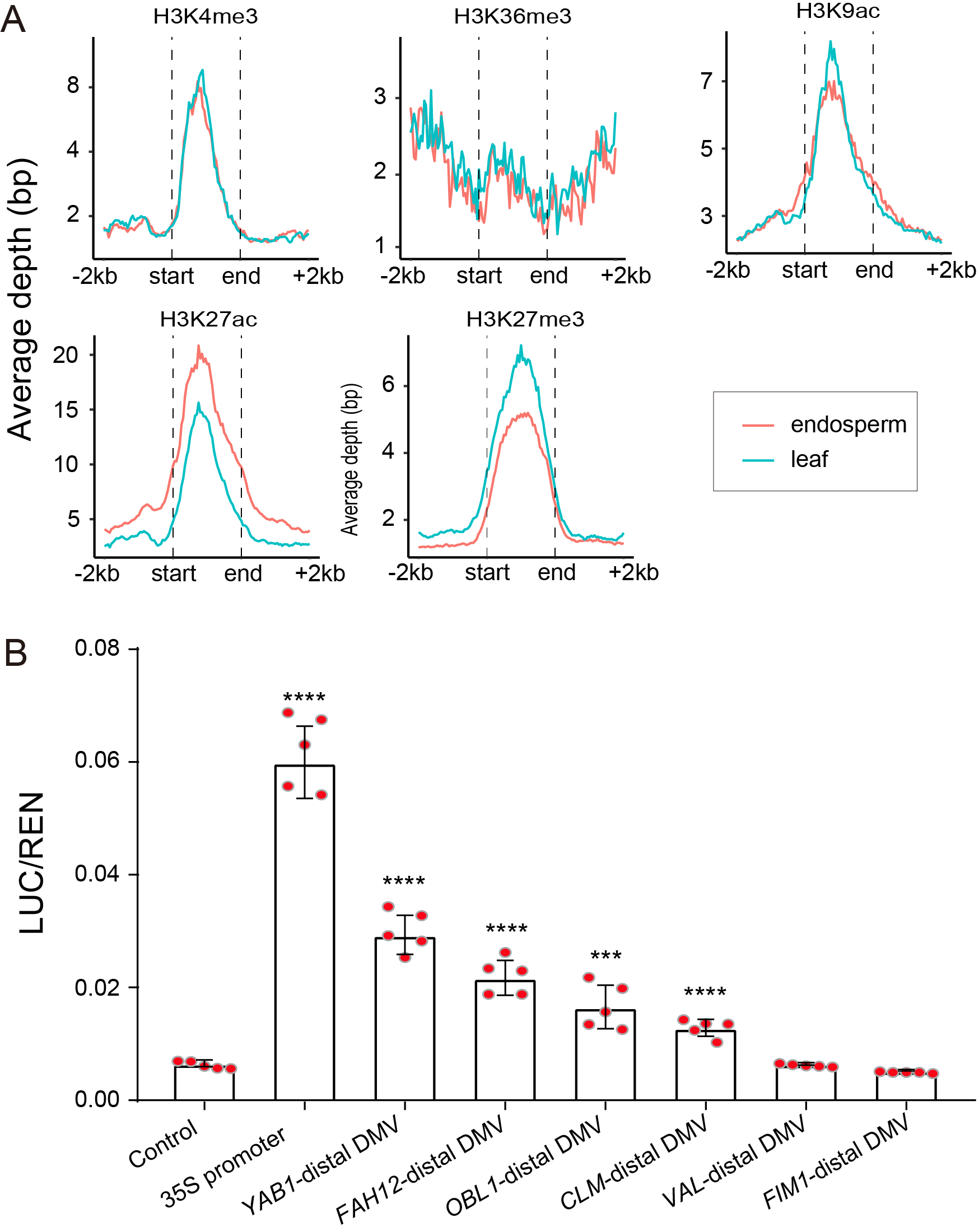


**Fig. S9. Changes of histone modifications over those distal DMVs that is near seed-specific genes and experimental validation of distal DMVs as enhancer by the dual-luciferase reporter assay system in *N. benthamiana* protoplasts.**

1. The average enrichment of histone modifications over those distal DMVs that is near seed-specific genes in leaf (red line) and endosperm (blue line). The read counts of ChIP-seq for each histone modifications is averaged over DMVs and the upstream and downstream 2kb regions.
2. The expression ratios of LUC/REN luciferase. Error bars indicated SDs from five biological replicates. Statistical significance was determined by a two-sided t-test. Six distal DMV of seed-specific genes were used for experimental analysis, including *YAB1* (28200.m000191), *FAH12* (28035.m000362), *OBL1* (29935.m000048), *CLM* (30005.m001270), *VAL* (29626.m000155) and *FIM1* (29854.m001125).
